# Original Chemical Series of Pyrimidine Biosynthesis Inhibitors That Boost the Antiviral Interferon Response

**DOI:** 10.1128/AAC.00383-17

**Published:** 2017-09-22

**Authors:** Marianne Lucas-Hourani, Daniel Dauzonne, Hélène Munier-Lehmann, Samira Khiar, Sébastien Nisole, Julien Dairou, Olivier Helynck, Philippe V. Afonso, Frédéric Tangy, Pierre-Olivier Vidalain

**Affiliations:** a Unité de Génomique Virale et Vaccination, Institut Pasteur, CNRS UMR3569, Paris, France; b Institut Curie, Centre de Recherche, CNRS UMR3666, INSERM U1143, Paris, France; c Unité de Chimie et Biocatalyse, Institut Pasteur, CNRS UMR3523 Paris, France; d IRIM CNRS UMR9004, Université de Montpellier, Montpellier, France; e Equipe Chimie Bio-inorganique des Dérivés Soufrés et Pharmacochimie, Université Paris Descartes, CNRS UMR8601, Paris, France; f Unité d'Epidémiologie et Physiopathologie des Virus Oncogènes, Département de Virologie, Institut Pasteur, CNRS UMR3569, Paris, France; g Equipe Chimie & Biologie, Modélisation et Immunologie pour la Thérapie (CBMIT), Université Paris Descartes, CNRS UMR8601, Paris, France

**Keywords:** antiviral agents, innate immunity, interferons, pyrimidine metabolism

## Abstract

*De novo* pyrimidine biosynthesis is a key metabolic pathway involved in multiple biosynthetic processes. Here, we identified an original series of 3-(1*H*-indol-3-yl)-2,3-dihydro-4*H*-furo[3,2-*c*]chromen-4-one derivatives as a new class of pyrimidine biosynthesis inhibitors formed by two edge-fused polycyclic moieties. We show that identified compounds exhibit broad-spectrum antiviral activity and immunostimulatory properties, in line with recent reports linking *de novo* pyrimidine biosynthesis with innate defense mechanisms against viruses. Most importantly, we establish that pyrimidine deprivation can amplify the production of both type I and type III interferons by cells stimulated with retinoic acid-inducible gene 1 (RIG-I) ligands. Altogether, our results further expand the current panel of pyrimidine biosynthesis inhibitors and illustrate how the production of antiviral interferons is tightly coupled to this metabolic pathway. Functional and structural similarities between this new chemical series and dicoumarol, which was reported before to inhibit pyrimidine biosynthesis at the dihydroorotate dehydrogenase (DHODH) step, are discussed.

## INTRODUCTION

Pyrimidine nucleosides, which include uridine, cytidine, and thymidine, are paramount precursors for DNA and RNA synthesis. They also play a pivotal role as intermediates in the synthesis of membrane glycerophospholipids, glycosylated proteins, and glycogen. UDP and triphosphate are also well-known extracellular mediators that signal through P2Y membrane receptors. Altogether, this makes pyrimidine nucleosides essential to cellular metabolism. Cells rely on three sources to fulfill their needs in pyrimidines. First, the so-called “salvage pathway” recycles pyrimidine nucleobases and nucleosides produced by the degradation of RNA and DNA molecules. In addition, cells often express passive and active transmembrane transporters that enable the uptake of pyrimidines, and in particular uridine, from extracellular body fluids (1). Finally, the *de novo* biosynthesis pathway, which allows the production of pyrimidines from glutamine, aspartate, and bicarbonate, is essential for proliferating cells to meet their large demand for nucleotide precursors (2). On the contrary, the salvage pathway combined to pyrimidine absorption from the extracellular pool is usually sufficient to satisfy the needs of nondividing cells. A multifunctional protein called CAD catalyzes the initial steps of *de novo* pyrimidine biosynthesis by physically linking three enzymes: the carbamoyl-phosphate synthetase (CPSase), the aspartate transcarbamylase (ATCase), and the dihydroorotase (DHOase). The fourth enzymatic step is catalyzed by the dihydroorotate dehydrogenase (DHODH), which is bound to the inner membrane of mitochondria, where it converts dihydroorotate (DHO) to orotate (3). Finally, the multifunctional UMP synthase uses orotate to produce UMP, a common precursor of all other pyrimidine nucleosides.

It has been recently shown that compounds inhibiting the *de novo* pyrimidine biosynthesis pathway exhibit potent broad-spectrum antiviral activity (4–11). Indeed, several screening campaigns for antiviral molecules led to the identification of either CAD or DHODH inhibitors. Such molecules were found to efficiently block the *in vitro* replication of many viruses, including both DNA and RNA viruses. In the presence of pyrimidine biosynthesis inhibitors, cellular pools of pyrimidines collapse, and the lack of pyrimidine is usually considered to be directly responsible for the inhibition of viral growth. However, it was also reported that inhibiting *de novo* pyrimidine biosynthesis stimulates the innate immune response, in particular the transcription of some interferon-stimulated genes (ISGs) independently of interferons (IFNs) and the canonical JAK-STAT pathway (8, 12–18). In addition, the antiviral activity of pyrimidine biosynthesis inhibitors was found to be strictly dependent on cellular gene transcription and nuclear export machinery and required interferon regulatory factor 1 (IRF1), a key transcription factor driving the expression of antiviral genes, including ISGs (8). More recently, it was shown that pyrimidine biosynthesis inhibitors could increase the expression of retinoic acid-inducible gene 1 (RIG-I), a cytoplasmic sensor inducing the expression of innate immunity genes and IFNs in response to RNA virus infections (16). Altogether, these different reports support a key role of the innate immune response in the antiviral activity of compounds inhibiting the *de novo* pyrimidine biosynthesis pathway. However, the mechanisms linking the intracellular pool of pyrimidines to the innate immune response remain to be characterized.

Here, we describe a novel series of 3-(1*H*-indol-3-yl)-2,3-dihydro-4*H*-furo[3,2-*c*]chromen-4-one derivatives inhibiting *de novo* pyrimidine biosynthesis. The lead molecule from this series, called DD363, was isolated from a screening campaign that was previously described and aimed at identifying stimulators of antiviral genes (8). The phenotypic assay we used was based on human HEK-293T cells transiently transfected with a luciferase reporter gene controlled by five interferon-stimulated response elements (ISRE). This regulatory element is present in promoter sequences of ISGs, where it binds transcription factors activated in type I interferon-stimulated or virus-infected cells, such as STAT1/STAT2/IRF9 (ISGF3) or IRFs. It was therefore expected that any compound inducing the ISRE-luciferase construct would also stimulate the expression of endogenous ISGs and exhibit some potent broad-spectrum antiviral activity. This phenotypic assay was used to screen a total of 41,353 chemical compounds for their capacity to stimulate ISRE-luciferase expression. Two compounds from the chemical library of Institut Curie were finally selected for further studies, including DD264, which has already been described (8), and DD363, which is novel in terms of structure and activity. Most interestingly, a functional study of this chemical series led us to show for the first time that in cells transfected with RIG-I ligands mimicking a viral infection, the production of type I interferon (IFN-I) and IFN-III is strongly boosted when *de novo* pyrimidine biosynthesis is blocked. This new observation unravels a mechanism by which cells modulate their communication with neighboring cells as a function of their metabolic status.

## RESULTS

### DD363 is an ISRE-luciferase-inducing antiviral compound.

**FIG 1:**
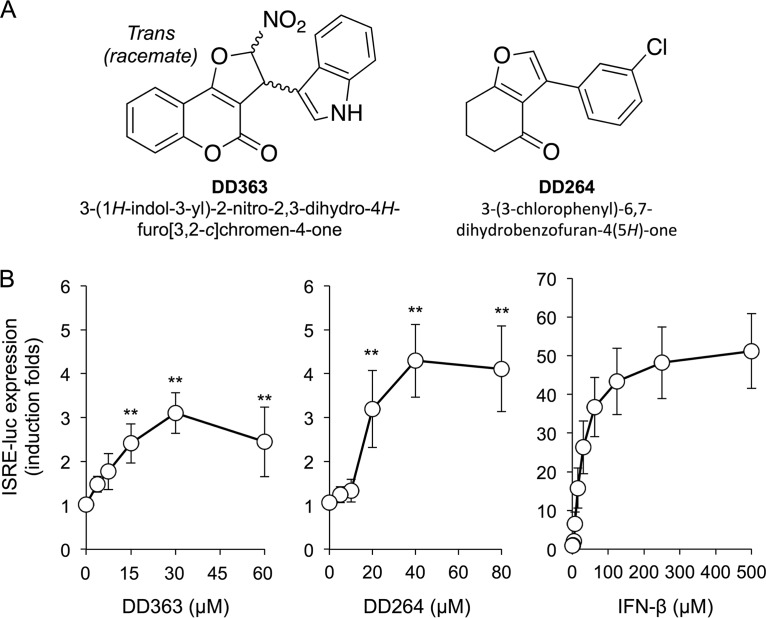
DD363 activates the ISRE-luciferase reporter gene. (A) Chemical structures of DD363 and DD264 from Lucas-Hourani et al. (8). (B) HEK-293 cells stably transfected with the ISRE-luciferase reporter gene (STING-37 cells) were incubated with increasing doses of DD363, DD264, or recombinant IFN-β. After 24 h, luciferase expression was determined and the results expressed as a fold change relative to that with DMSO. Data correspond to means ± SD of the results from 8, 5, and 7 independent experiments, respectively. **, *P* < 0.01 as calculated by one-way analysis of variance (ANOVA) with Bonferroni's *post hoc* test.

DD363 was first selected in a chemical screen because, compared to other tested molecules, it significantly stimulated the expression of the ISRE-luciferase reporter plasmid transiently transfected in HEK-293T cells (6.2-fold induction at 56 μM in the initial screen). DD363 is 3-(1*H*-indol-3-yl)-2,3-dihydro-4*H*-furo[3,2-*c*]chromen-4-one, which corresponds to a novel chemical series never reported before (Fig. 1A). It is different from DD264 that we previously identified in the same screen (8), although parts of the molecule exhibit similarities (Fig. 1A). To confirm DD363 activity, this compound was tested in a dose-response experiment on STING-37 cells, a reporter cell line derived from human HEK-293 cells by stable transfection of the ISRE-luciferase reporter gene. As shown in Fig. 1B, a 3-fold increase in luciferase expression was observed upon DD363 treatment. This is slightly inferior to the results obtained with DD264 but in a similar range. DD363 and DD264 are, however, weak inducers of the ISRE-luciferase reporter gene compared to recombinant beta interferon (IFN-β) (Fig. 1B). Nevertheless, we decided to further characterize this molecule and evaluate its antiviral activity in secondary assays.

**FIG 2:**
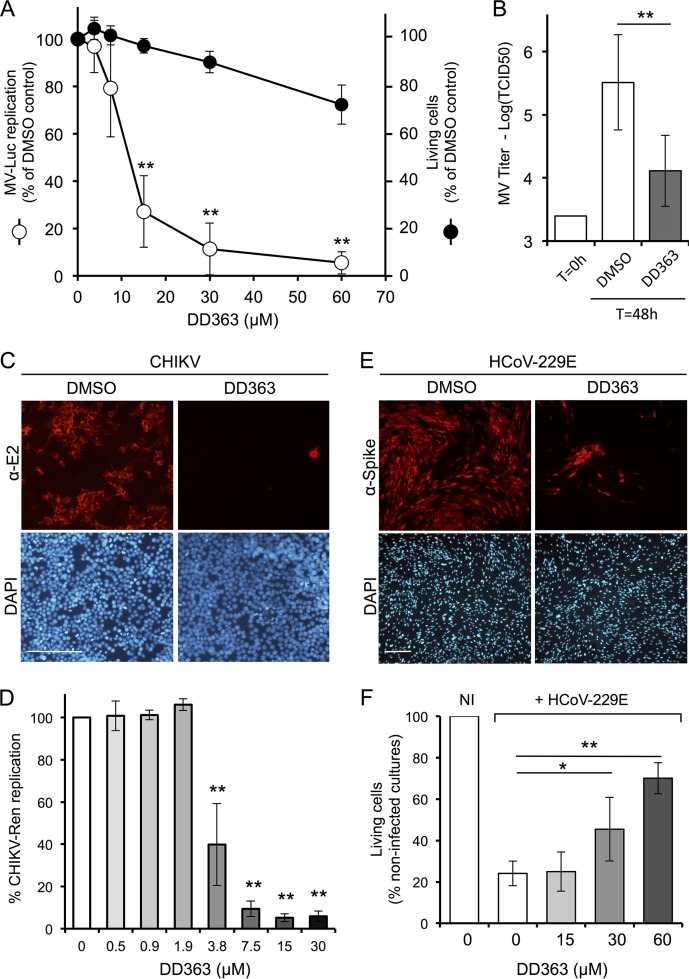
DD363 is an antiviral compound. (A) HEK-293T cells were infected with MV-Luc at a multiplicity of infection (MOI) of 0.1 and incubated with increasing concentrations of DD363 or DMSO alone. After 24 h, luciferase activity was measured to quantify viral growth (open circles). Results are expressed as a percentage of luminescence signals relative to a DMSO control (means ± SD of the results from 10 independent experiments). **, *P* < 0.01, as calculated by one-way ANOVA with Bonferroni's *post hoc* test. As a control, HEK-293T cells were treated with increasing concentrations of DD363 or DMSO alone. After 24 h, cellular viability was determined using CellTiter-Glo reagent (Promega), which evaluates by ATP quantification the number of metabolically active cells (closed circles). Results are expressed as a percentage relative to a DMSO control (means ± SD of the results from 4 independent experiments). (B) HEK-293T cells were infected with MV (MOI = 0.1) and incubated with DD363 (30 μM) or DMSO alone. At the time of infection (T = 0) and 2 days later (T = 48), cultures were harvested and virus titers determined by the TCID_50_ method (means ± SD of the results from 5 independent experiments). **, *P* < 0.01, as calculated by paired two-tailed *t* test. (C) HEK-293T cells were infected with CHIKV (MOI = 0.1) and incubated with DMSO alone or DD363 (30 μM). After 24 h, cells were fixed, and CHIKV E2 glycoprotein was detected by immunostaining. Cell nuclei were stained with DAPI. Scale bar = 200 μm. (D) HEK-293T cells were infected with a recombinant strain of CHIKV expressing Renilla luciferase (MOI = 0.2) and incubated with DD363 or DMSO alone. After 24 h, Renilla luciferase expression was determined. Results are expressed as a percentage relative to a DMSO control (means ± SD of the results from 3 independent experiments). **, *P* < 0.01, as calculated by one-way ANOVA with Bonferroni's *post hoc* test. (E) MRC5 cells were infected with HCoV-229E (MOI = 0.1) and incubated with DMSO alone or DD363 (60 μM). After 48 h, cells were fixed, and HCoV-229E spike glycoprotein was detected by immunostaining. Cell nuclei were stained with DAPI. Scale bar = 200 μm. (F) MRC5 cells were infected with HCoV-229E (MOI = 0.1) or not (noninfected [NI]) and incubated with DMSO alone or increasing concentrations of DD363. After 48 h, the number of viable cells in culture wells was determined using the CellTiter-Glo reagent (Promega) and normalized to the number of viable cells in noninfected DD363-treated cultures to take into account cytostatic effects of the molecule. Results correspond to means ± SD of 5 independent experiments. *, *P* < 0.05; and **, *P* < 0.01, as calculated by one-way ANOVA with Bonferroni's *post hoc* test.

At first, HEK-293T cells were infected with a recombinant strain of measles virus (MV) expressing luciferase as a reporter of viral replication (MV-Luc) and then treated with increasing concentrations of DD363 for 24 h. As shown in Fig. 2A, DD363 efficiently inhibited MV growth, as assessed by decreased levels of luciferase activity in cultures. The half-maximal inhibitory concentration (IC_50_) of DD363 (mean ± standard deviation [SD]) was estimated to be 11 ± 3 μM in this assay (Table 1). The inhibition of MV-Luc was persistent at 48 h postinfection but was partially lost at 72 h and limited to the highest concentration of DD363, suggesting that the virus progressively overcame the antiviral effects (see Fig. S1A in the supplemental material). We also showed that DD363 at 30 μM reduced by 1 log the production of infectious MV particles in cell cultures, as determined by virus titration at 48 h postinfection (Fig. 2B). Besides, this compound showed limited cytotoxicity at these concentrations (Fig. 2A), as determined by quantification of the number of living cells in DD363 versus dimethyl sulfoxide (DMSO)-treated wells after 24 h of culture. Under these conditions, the 50% cytotoxic concentration (CC_50_) of the molecule (mean ± SD) was estimated to 87 ± 7 μM. However, kinetic studies showed that DD363 essentially inhibited cellular proliferation rather than killed cells at concentrations below 60 μM. Indeed, the number of viable cells increased less rapidly in DD363-treated cultures but did not collapse below the initial number of cells seeded at T = 0 h (Fig. S1B). Furthermore, the fraction of dead cells in cultures did not increase from 0 to 72 h of culture, and it always remained below 15% in the presence or absence of DD363, as assessed by flow cytometry analysis (LIVE/DEAD fixable green dead cell stain kit; data not shown).

**TABLE 1:**
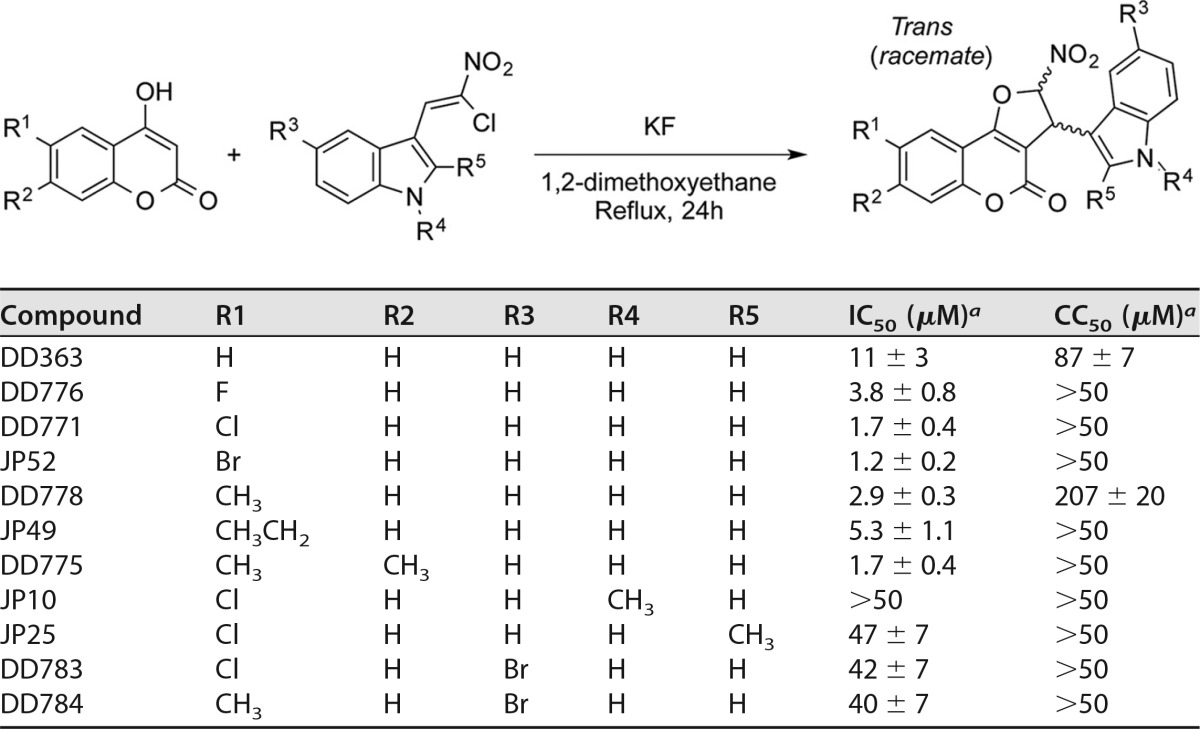


a Means ± SD of the results from ≥3 independent experiments. IC_50_ was determined by quantifying the inhibition of MV-Luc in HEK-293T cells after 24 h of culture. CC_50_ was determined with the CellTiter-Glo reagent (Promega) in noninfected HEK-293T cells after 24 h of culture.

Then, DD363 antiviral activity was tested on two other RNA viruses: chikungunya virus (CHIKV) and human coronavirus 229E (HCoV-229E). HEK-293T cells were infected with CHIKV and treated with DD363 or DMSO alone for 24 h, and cells were immunostained for the expression CHIKV glycoprotein E2. As shown in Fig. 2C, DD363 efficiently suppressed CHIKV replication in these cells. Inhibition by DD363 was also confirmed by the infection of HEK-293T cells with a recombinant strain of CHIKV expressing Renilla luciferase as a reporter of viral growth (Fig. 2D; mean ± SD IC_50_ = 3.6 ± 0.6 μM) (19). Similarly, DD363 was found to inhibit HCoV-229E growth in human MRC5 cells (Fig. 2E). We also showed that DD363 reduced the cytopathic effects of HCoV-229E, as assessed by quantifying the number of viable cells in infected MRC5 cultures (Fig. 2F). Altogether, this demonstrated that DD363 is active against different negative-strand (MV) or positive-strand (CHIKV and HCoV-229E) RNA viruses, while it also inhibits cellular proliferation.

### Structure-activity analysis of DD363 analogs.

**FIG 3:**
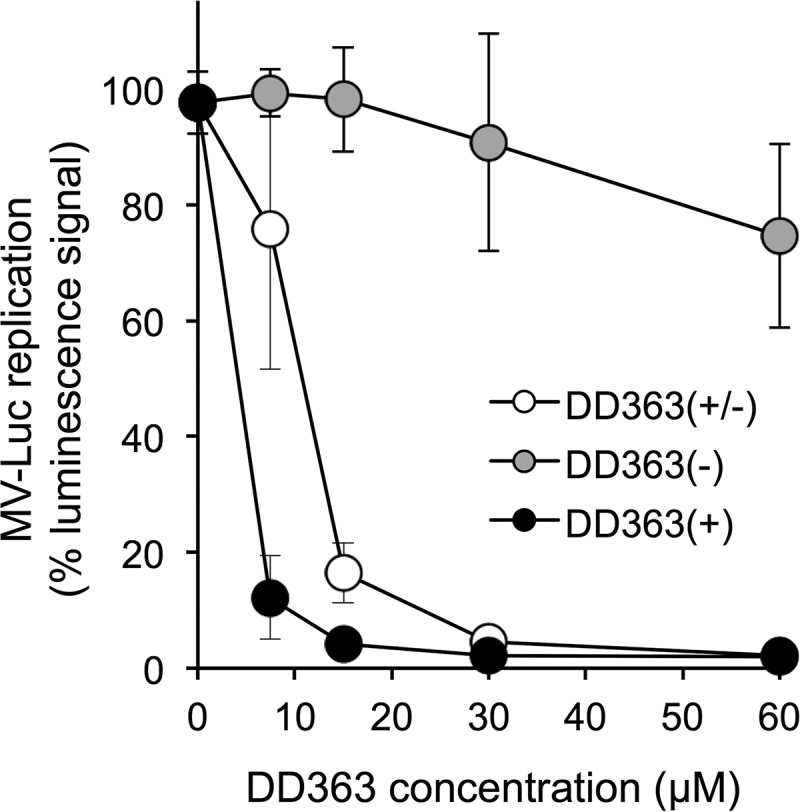
Antiviral activity of DD363 isolated enantiomers. Enantiomers of DD363 were separated by chiral chromatography and then tested for the inhibition of MV-Luc in HEK-293T cells as in Fig. 2A. The (±) symbol indicates a mixture of enantiomers, whereas (+) and (−) symbols correspond to purified enantiomers. Data correspond to means ± SD of the results from 5 independent experiments.

To characterize structural features that account for DD363 activity, we first decided to separate the two enantiomers from this racemic mixture, as they could differ in their antiviral potencies. Thus, enantiomers of DD363 were separated by chiral chromatography and tested on HEK-293T cells infected by MV-Luc. Interestingly, only one of the two enantiomers (+) exhibited antiviral potency (Fig. 3). Altogether, this demonstrated that there are spatial requirements with respect to the connection between the indole moiety and the 2,3-dihydro-4*H*-furo[3,2-*c*]chromen-4-one.

**FIG 4:**
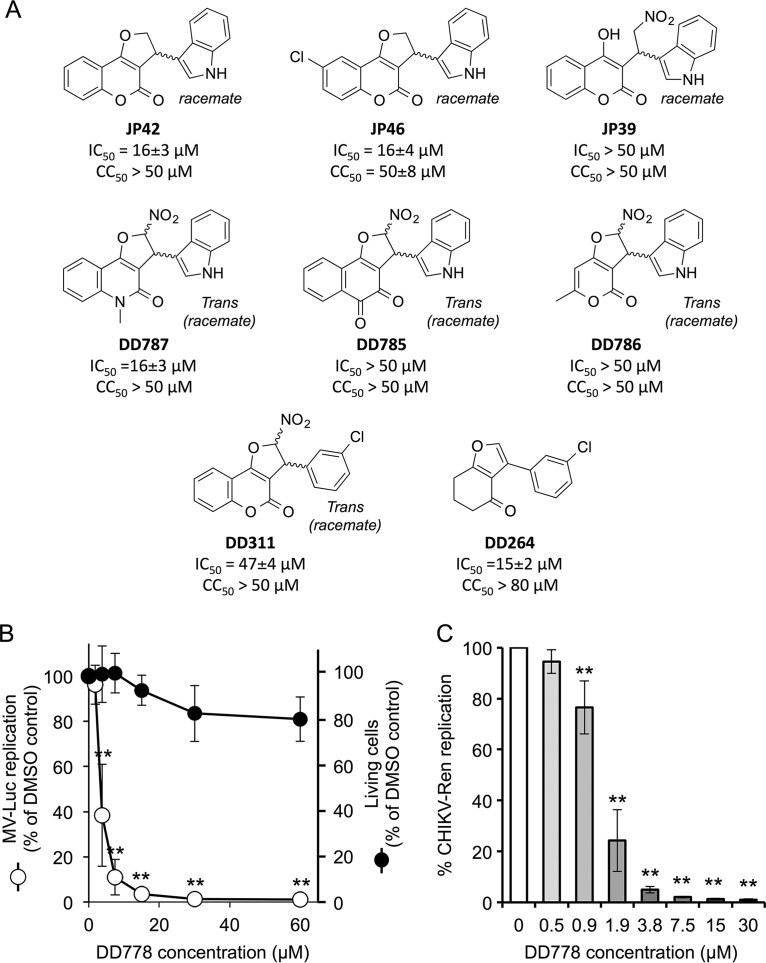
Antiviral activity of DD363 analogs, including DD778. (A) Increasing concentrations of DD363 analogs were tested for their capacity to inhibit MV-Luc in HEK-293T cells, and IC_50_s were calculated from dose-response curves. In parallel, the half-maximal cytotoxic concentration (CC_50_) was determined using CellTiter-Glo (Promega). Data correspond to means ± SD of the results from ≥3 independent experiments. (B) Dose-response curve showing the inhibition of MV-Luc (MOI = 0.1) by DD778 in HEK-293T cells after 24 h of culture (open circles). Results are expressed as a percentage of luminescence signals relative to DMSO control (means ± SD of the results from 3 independent experiments). **, *P* < 0.01, as calculated by one-way ANOVA with Bonferroni's *post hoc* test. In parallel, cellular viability in noninfected cultures treated with DD778 was determined with the CellTiter-Glo reagent (closed circles; means ± SD of the results from 4 independent experiments). (C) HEK-293T cells were infected with a recombinant strain of CHIKV expressing Renilla luciferase (MOI = 0.2) and incubated with increasing doses of DD778 or DMSO alone. After 24 h, Renilla luciferase expression was determined. Results are expressed as a percentage relative to DMSO control (means ± SD of the results from 3 independent experiments). **, *P* < 0.01, as calculated by one-way ANOVA with Bonferroni's *post hoc* test.

To further identify chemical features accounting for DD363 potency and select for more potent molecules, a set of 17 structural analogs was synthesized (see supplemental material) (20, 21). The antiviral potencies of these compounds were tested on HEK-293T cells infected by MV-Luc, and the corresponding IC_50_s are reported in Table 1 and Fig. 4A. Interestingly, the addition of a halogen group (DD776, DD771, and JP52), a methyl (DD778) group, or an ethyl group (JP49) at position R1 increased the antiviral potency (2- to 9-fold higher than for DD363; Table 1). As a control, DD778 was shown to inhibit not only MV (Fig. 4B and Table 1) but also CHIKV (Fig. 4C; IC_50_ = 1.4 ± 0.2 μM) replication, with an increased potency compared to DD363 (Fig. 2D). Then, highly potent analogs with a halogen group (DD771) or a methyl group (DD778) at position R1 were further modified at position R2, R3, R4, or R5 (Table 1). The addition of a methyl group at position R2 showed no major effect on the antiviral potency (DD775). In contrast, all compounds modified on the indole moiety were much less potent (JP10, JP25, DD783, and DD784), suggesting that this part of the molecule is essential (Table 1). We have also tested analogs of DD363 and DD771 without a nitro group on the furan ring (Fig. 4A; JP42 and JP46, respectively). Although DD363 and JP42 showed comparable antiviral potencies, JP46 was much less potent than DD771 (9-fold decrease). Besides, opening the furan ring (JP39) led to some loss of the antiviral potency, supporting the role played by this part of the molecule (Fig. 4A). Other structural modifications were tested on 2,3-dihydro-4*H*-furo[3,2-*c*]chromen-4-one, showing no benefit (DD787) or abrogating the antiviral potency (DD785 and DD786; Fig. 4A). Furthermore, replacement of the indole moiety group by a 3-chlorophenyl group (DD311; previously described in reference 20), as in DD264 (8), greatly decreased the antiviral activity by comparison with DD363 (Fig. 4A). Altogether, this established structure-activity relationships for this chemical series, and analogs that are up to 9-fold more potent than the initial hit DD363 were identified.

### DD778 is an inhibitor of pyrimidine biosynthesis.

As mentioned above, DD363 was selected from a screening campaign together with DD264, a compound subsequently characterized as an inhibitor of *de novo* pyrimidine biosynthesis (8). Although DD363 and its active analogs are chemically unrelated to DD264, they have similar antiviral properties and capacities to induce the ISRE-luciferase reporter gene. This suggested a similar mode of action. We thus determined whether DD778, one potent analog of DD363 showing a better selectivity index, could inhibit *de novo* pyrimidine biosynthesis like DD264.

**FIG 5:**
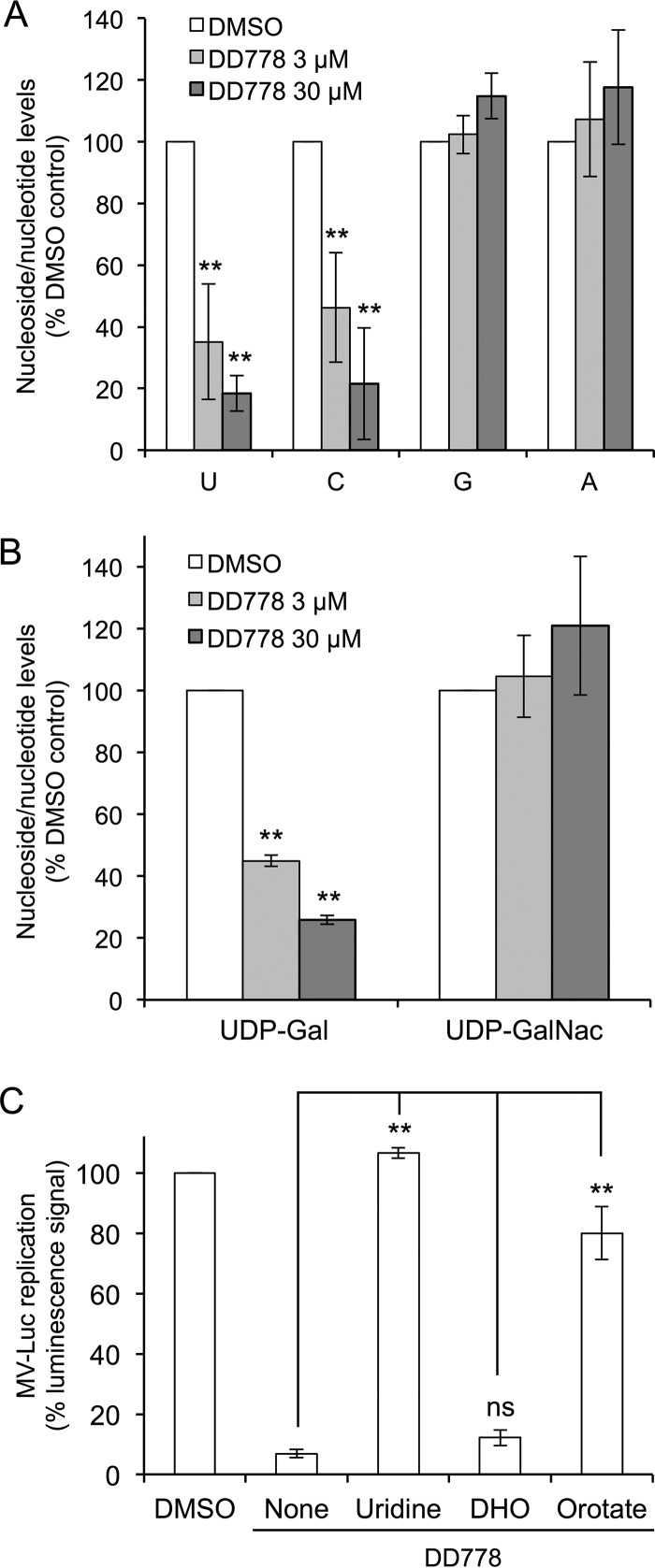
DD778 is an inhibitor of pyrimidine biosynthesis. (A) HEK-293T cells were treated for 24 h with DD778 or DMSO alone. Cells were harvested and washed in PBS, and intracellular levels of each nucleoside/nucleotide (U, C, G, or A) were determined by HPLC and spectrophotometry. Concentrations are expressed as a percentage relative to DMSO-treated cells (means ± SD of the results from 4 independent experiments). (B) Same as in panel A, but intracellular levels of UDP galactose (UDP-Gal) and UDP *N*-acetylgalactosamine (UDP-GalNac) are presented. Data represent means ± SD of the results from 3 independent experiments. (C) HEK-293T cells were infected with MV-Luc (MOI = 0.1), incubated with DMSO or DD778 (10 μM), and cotreated with uridine (130 μM), dihydroorotate (DHO; 3 mM) or orotate (3 mM). After 24 h, luciferase expression that reflects viral growth was determined. Data represent means ± SD of the results from 3 independent experiments. **, *P* < 0.01, as calculated by one-way ANOVA with Bonferroni's *post hoc* test. ns, nonsignificant.

To address this question, HEK-293T cells were treated with DD778 or DMSO alone, and nucleoside levels in cellular extracts were determined by high-performance liquid chromatography (HPLC) and spectrophotometry analysis. As shown in Fig. 5A, uridine (U) and cytidine (C) levels collapsed in DD778-treated cells, whereas purine levels (A and G) were unaffected. This demonstrated that DD778 interferes with pyrimidine metabolism and homeostasis. Of its multiple functions, uridine is also required to synthesize UDP-sugar complexes, which are essential to protein glycosylation. We thus determined whether DD778 could also alter intracellular levels of UDP-galactose (UDP-Gal) and UDP *N*-acetylgalactosamine (UDP-GalNac). UDP-Gal collapsed in DD778-treated cells, as predicted. Surprisingly, UDP-GalNac was maintained at high levels, suggesting that specific mechanisms help secure intracellular concentrations of this metabolite (Fig. 5B).

We then determined if the lack of pyrimidine nucleosides in DD778-treated cells accounts for the antiviral potency of this compound. Thus, HEK-293T cells were infected with MV-Luc and then cultured with DD778 in a medium supplemented with either uridine or precursors of pyrimidine nucleoside biosynthesis, including dihydroorotate (DHO) or orotate (Fig. 5C). Uridine and orotate were found to restore viral replication in DD778-treated cells, as assessed by luciferase expression, whereas DHO showed no effect. This established that low levels of pyrimidine nucleosides account for the inhibition of viral growth in DD778-treated cells. Furthermore, the fact that orotate but not DHO could revert the antiviral effects of DD778 suggested that DHODH, the fourth enzyme of pyrimidine biosynthesis that converts DHO to orotate, is the target of DD778 (Fig. 5C). This is in line with previous reports, including ours, showing that DHODH is the target of numerous broad-spectrum antiviral compounds (4–18).

### DD778 amplifies interferon expression in cells stimulated with RIG-I ligands.

**FIG 6:**
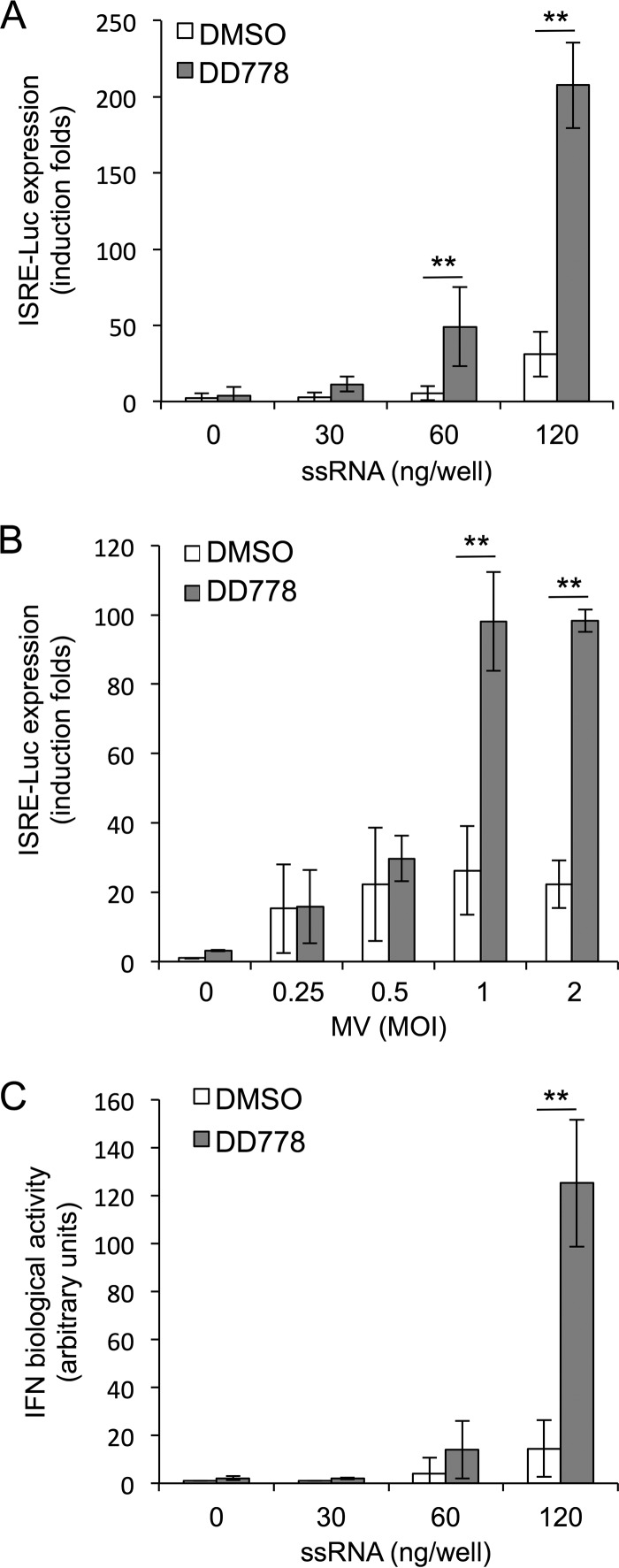
Cellular response to ssRNA transfection is amplified by DD778. (A) A total of 2 × 10^5^ STING-37 cells were transfected with indicated doses of ssRNA and immediately treated with DMSO alone or DD778 (30 μM). After 24 h, luciferase activity was determined. Data represent means ± SD of the results from 5 independent experiments. **, *P* < 0.01, as calculated by two-way ANOVA with Bonferroni's *post hoc* test. (B) STING-37 cells were infected with MV at the indicated MOI and cultured for 9 h. Then, medium was supplemented with DD778 (30 μM) or DMSO alone, and luciferase expression was determined 16 h later. Data represent means ± SD of the results from 6 independent experiments. **, *P* < 0.01, as calculated by two-way ANOVA with Bonferroni's *post hoc* test. (C) Same as in panel A, but culture supernatants were harvested at T = 48 h, and IFN biological activity was determined on fresh STING-37 reporter cells. Data represent means ± SD of the results from 5 independent experiments. **, *P* < 0.01, as calculated by two-way ANOVA with Bonferroni's *post hoc* test.

We investigated potential interactions between DD778 and the innate antiviral response, in line with previous reports showing that inhibitors of *de novo* pyrimidine biosynthesis stimulate the expression of ISGs. First, it was confirmed that DD778 induced the ISRE-luciferase gene in STING-37 cells, like DD363, whereas the chemical analog DD786 that did not inhibit MV replication (Table 1) was unable to do so (Fig. S2A). We then determined if, as expected, DD778 could also amplify the cellular response to synthetic ligands of RIG-I that mimic viral nucleic acids from RNA viruses (22). STING-37 reporter cells were transfected with low doses of short synthetic 5′-triphosphate RNA molecules (ssRNA) to prime the innate immune response without reaching saturation and were cultured with DD778 or DMSO alone for 24 h. As shown in Fig. 6A, DD778 amplified ISRE-luciferase expression in response to ssRNA transfection. In contrast, DD786 had no effect on cellular response toward ssRNA (Fig. S2B). We also tested the effects of DD778 on ISRE-luciferase induction by a virus. Thus, STING-37 cells were infected with MV at different multiplicities of infection (MOIs) and incubated for 9 h to let the virus trigger the innate immune response, and DD778 or DMSO alone was added for an additional incubation period of 16 h. As shown in Fig. 6B, DD778 efficiently boosted the cellular response to MV infection, as assessed by increased expression levels of the ISRE-luciferase reporter gene. Finally, we determined if DD778 could also boost the expression of IFNs in response to ssRNA, something that was not investigated before. To address this question, supernatants from cell cultures transfected with ssRNA and treated with DD778 or DMSO alone for 48 h were collected, diluted twice, and applied to fresh STING-37 reporter cells to measure IFN biological activity with the ISRE-luciferase reporter gene. As shown in Fig. 6C, DD778 strongly amplified the IFN activity in culture supernatants of ssRNA-transfected cells. It should be noticed that the supernatants of cells treated with DD778 alone did not show any IFN activity, in line with previous reports using other pyrimidine biosynthesis inhibitors (8, 16).

**FIG 7:**
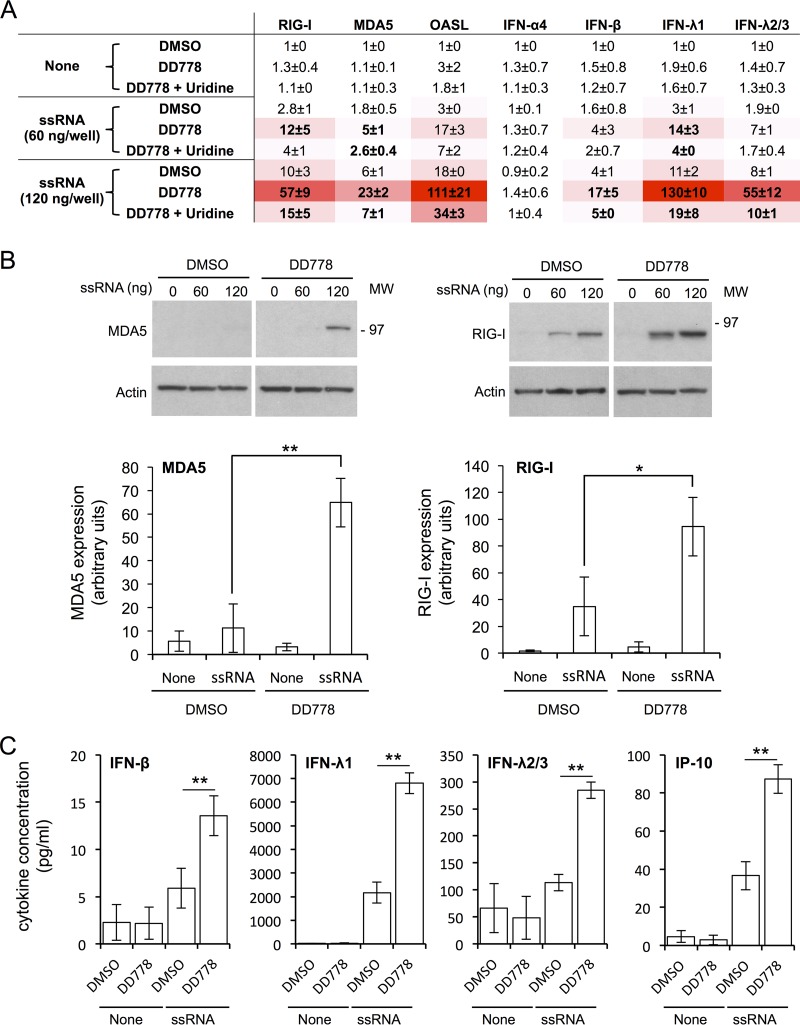
The expression of ISGs and IFNs in ssRNA-stimulated cells is enhanced by DD778. (A) HEK-293 cells with the ISRE-luciferase reporter gene (STING-37) were transfected with indicated doses of ssRNA and immediately treated with DMSO alone or DD778 (30 μM). Total RNAs were extracted from cellular pellets collected at T = 24 h, and the expression levels of specified genes were determined by RT-qPCR. Data represent means ± SD of the results from 3 independent experiments. Bold figures correspond to statistically significant differences comparing DD778 to DMSO-treated samples or DD778 + uridine to DD778 alone (*P* < 0.05, calculated by two-way ANOVA with Bonferroni's *post hoc* test). (B) Same as above, but MDA5 and RIG-I expression levels were determined by Western blotting on total protein extracts from STING-37 cells at T = 48 h. Upper images correspond to representative experiments. Graphs below correspond to the quantification of Western blotting results, where values were normalized to actin (means ± SD of the results from 3 independent experiments; cells were transfected with 120 ng of ssRNA). *, *P* < 0.05; and **, *P* < 0.01, as calculated by one-way ANOVA with Bonferroni's *post hoc* test. (C) Same experiment as above, but total concentrations of indicated cytokines in culture supernatants at T = 48 h were determined using the LEGENDplex analysis kit (cells were transfected with 120 ng of ssRNA). Data represent means ± SD of the results from 4 independent experiments. *, *P* < 0.05; and **, *P* < 0.01, as calculated by two-tailed standard *t* test.

To further document the consequences of DD778 treatment on the cellular response to ssRNA, mRNA levels for RIG-I, MDA5, oligoadenylate synthase-like protein (OASL), IFN-α4, IFN-β, IFN-λ1, and IFN-λ2/3 were determined by reverse transcription-quantitative PCR (RT-qPCR). The results showed that in response to ssRNA transfection, DD778 amplified the induction of RIG-I, MDA5, OASL, IFN-β, IFN-λ1, and IFN-λ2/3 (Fig. 7A). Furthermore, the effects of DD778 on the expression of these genes could be reversed by the addition of uridine in culture medium. This demonstrated that pyrimidine depletion is responsible for the amplified response to ssRNA, in agreement with our previous report on DD264 (8). To confirm these results at the protein level, we determined MDA5 and RIG-I expression by Western blotting. As shown in Fig. 7B, DD778 promoted the expression of MDA5 and RIG-I in cells transfected with ssRNA. In parallel, we measured in culture supernatants the concentrations of IFN-β, IFN-λ1, IFN-λ2/3, and interferon-inducible protein of 10 kDa (IP-10), which is a well-characterized IFN-inducible chemokine (Fig. 7C). We found that DD778 also increased the expression of IFN-β, IFN-λ1, IFN-λ2/3, and IP-10 when cells were stimulated with ssRNA. Altogether, our results showed that DD778 amplifies the expression of ISGs in cells stimulated with ssRNA but also produced higher levels of type I and III IFNs. This illustrates an original mechanism by which cells can communicate with neighbors on their metabolic status.

### DD778 modulates the innate immune response in MV-infected cells.

**FIG 8:**
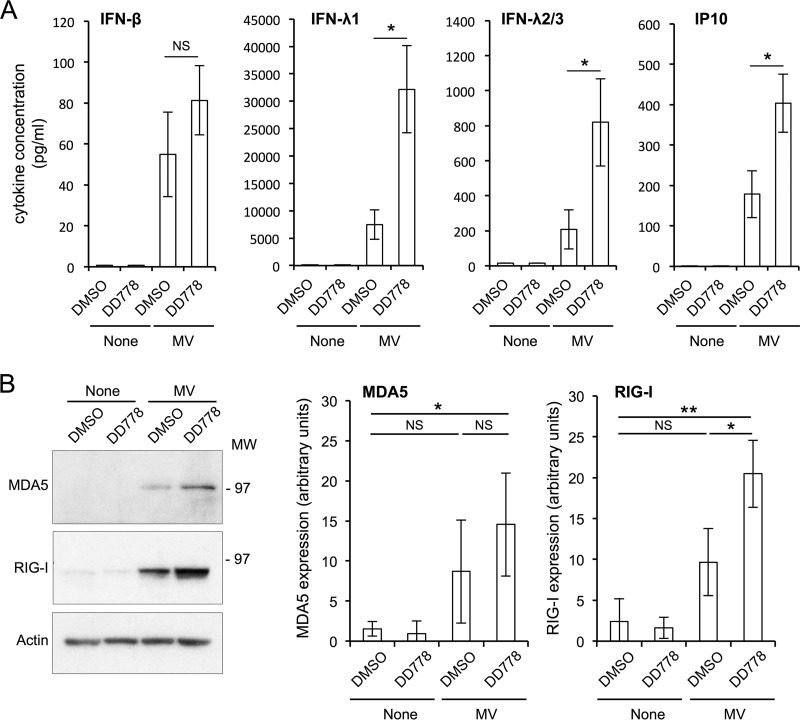
DD778 modulates the innate immune response in MV-infected cells. (A) STING-37 cells were infected with MV (MOI = 2) and cultured for 9 h. Then, medium was supplemented with DD778 (30 μM) or DMSO alone. Culture supernatants and cells were harvested 39 h later. Total concentrations of indicated cytokines in culture supernatants were determined using the LEGENDplex analysis kit. Data represent means ± SD of the results from 3 independent experiments. *, *P* < 0.05, as calculated by two-tailed standard *t* test. (B) Same experiment as above, but MDA5 and RIG-I expression levels were determined by Western blotting on cell protein extracts. Left images correspond to one representative experiment. Right graphs correspond to the quantification of Western blotting results, where values were normalized to actin (means ± SD of the results from 4 independent experiments). *, *P* < 0.05; and **, *P* < 0.01, as calculated by one-way ANOVA with Bonferroni's *post hoc* test.

Finally, we determined if DD778 also amplified the expression of IFN-β, IFN-λ1, IFN-λ2/3, IP-10, MDA5, and RIG-I in the context of a viral infection. STING-37 cells were infected with MV (MOI = 2) and incubated for 9 h to let the virus trigger the innate immune response, and DD778 or DMSO alone was added for an additional incubation period of 39 h. IFN-β, IFN-λ1, IFN-λ2/3, and IP-10 were quantified in culture supernatants (Fig. 8A), whereas MDA5 and RIG-I expression levels were determined by Western blotting on cellular pellets (Fig. 8B). As shown in Fig. 8A, MV alone induced the different cytokines, and DD778 efficiently increased the expression of IFN-λ1, IFN-λ2/3, and IP-10. The expression of RIG-I in MV-infected cells was also boosted in the presence of DD778. Surprisingly, IFN-β expression in MV-infected cells was not statistically enhanced by DD778 treatment (Fig. 8A), in contrast to results obtained with ssRNA (Fig. 7C). The effects on MDA5 expression were also limited, even though we observed a significant induction of MDA5 by MV plus DD778, but not with MV infection alone. One explanation is that MV encodes virulence factors that interfere with the innate immune response, in particular the V protein that binds directly to MDA5 to prevent its activation and counteract IFN-β expression (23–25). Nevertheless, the results obtained for RIG-I, IFN-λ1, IFN-λ2/3, and IP-10 clearly demonstrate that DD778 can boost the innate immune response in virus-infected cells, in particular the expression of type III IFNs, which were produced at high levels in this system.

## DISCUSSION

**FIG 9:**
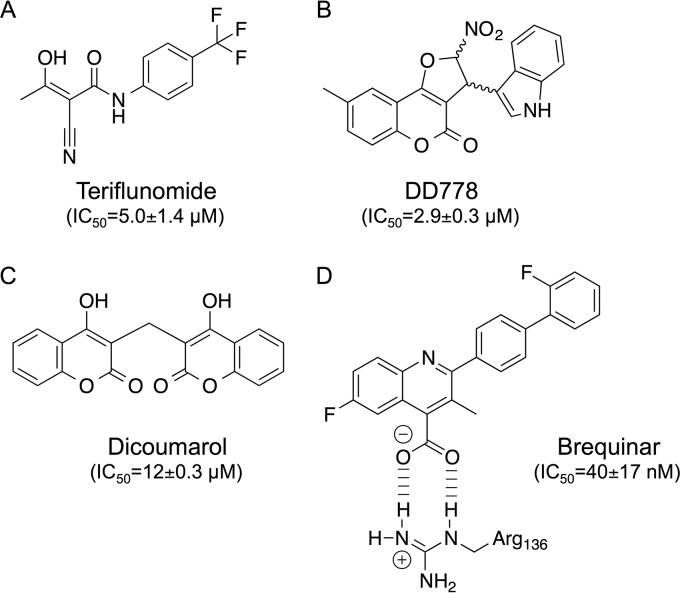
Chemical structures of known DHODH inhibitors compared to DD778. (A to D) Chemical structures of teriflunomide (A), DD778 (B), dicoumarol (C), and brequinar (D). (D) Interactions with Arg136 from human DHODH (based on 1D3G [35]). Indicated IC_50_s correspond to the inhibition of MV-Luc in HEK-293T cells, as determined in this paper for DD778 and dicoumarol, or as previously reported by Munier-Lehmann et al. (9) for teriflunomide and brequinar.

Components of the *de novo* pyrimidine biosynthesis pathway, and DHODH in particular, are becoming drug targets of prime interest in the treatment of multiple pathologies, including viral and microbial infections, cancer, and autoimmune diseases (3). A most recent example is a breakthrough study showing the therapeutic potential of DHODH inhibitors in the treatment of acute myeloid leukemia (26). So far, teriflunomide and its prodrug leflunomide, which are used as immunosuppressors in the treatment of multiple sclerosis and rheumatoid arthritis, are the only two DHODH inhibitors on the market (Fig. 9). However, it has been shown that the immunosuppressive properties of these drugs and their capacity to inhibit lymphocyte proliferation *in vivo* rely on interactions with secondary targets, and in particular tyrosine kinases (27). Therefore, the identification of highly active and specific inhibitors of *de novo* pyrimidine biosynthesis is a field of intense competition. In the last decade, many lead compounds that are mostly targeting DHODH have been characterized in detail and are currently at a different stage of development. However, these molecules can be clustered in a relatively limited number of chemical families, and there is still a great need for original structures that could help in the development of molecules suitable for the clinic (3).

In this study, we describe a new class of pyrimidine biosynthesis inhibitors based on 3-(1*H*-indol-3-yl)-2,3-dihydro-4*H*-furo[3,2-*c*]chromen-4-one. Complementation experiments in cell-based assays strongly suggest that DD778 and related analogs target DHODH, but this will require confirmation by crystallography or enzymatic studies. We previously determined the antiviral potencies of brequinar and teriflunomide in HEK-293T cells infected by MV-Luc, and IC_50_s were estimated at 40 nM and 5 μM, respectively (Fig. 9). Therefore, the antiviral potency of DD778 in this assay is comparable to that of teriflunomide but is much lower than that of brequinar or other DHODH inhibitors, such as the series of 2-(3-alkoxy-1*H*-pyrazol-1-yl)pyrimidines that we recently described (IC_50_ = 0.7 nM for 21q) (9). Accordingly, brequinar induced the ISRE-luciferase reporter gene in STING-37 cells and inhibited *de novo* pyrimidine biosynthesis at submicromolar concentrations (Fig. S3A and B). Thus, DD778 and other tested analogs are clearly not among the most active DHDOH inhibitors, but their interest lies in their chemical structure. Indeed, these compounds are devoid of the carboxylic group characterizing DHODH inhibitors from the brequinar family, but they are also missing the amide group that is central to teriflunomide and its derivatives (Fig. 9). Furthermore, the novel chemical series described herein is based on two edge-fused polycyclic moieties linked together, something relatively unique among pyrimidine biosynthesis inhibitors described in the literature (3). To our knowledge, the only known DHODH inhibitor formed from two edge-fused polycyclic moieties is dicoumarol (IC_50_ = 6.6 μM when tested on the recombinant enzyme) (28), but the description of its activity was limited, and no effect on innate immunity or viral growth has been reported before (Fig. 9) (28, 29). We thus tested dicoumarol in our different assays and found that it induced the ISRE-luciferase reporter gene in STING-37 cells (Fig. S3C), amplified the cellular response to ssRNA transfection (Fig. S3D), and efficiently inhibited MV replication (IC_50_ = 12 ± 0.3 μM; Fig. S3E). Most importantly, and despite the fact that dicoumarol has multiple cellular targets and biological activities, the antiviral effect was fully reversed when supplementing the culture medium with uridine (Fig. S3F), thus establishing a functional link with the inhibition of pyrimidine biosynthesis. Quite interestingly, the 4-hydroxycoumarin group of dicoumarol is showing obvious similarities with the dihydrofurochromenone of DD778. Molecular docking experiments on DHODH structures also suggest that 4-hydroxycoumarin can be regarded as a substitute to the quinoline-4-carboxylic acid of brequinar to ensure critical interactions with Arg136 of DHODH (28). It is thus tempting to speculate that the 2,3-dihydro-4*H*-furo[3,2-*c*]chromen-4-one moiety of DD778 and other active analogs play the same role. Altogether, our findings expand our current knowledge of chemical possibilities to target the pyrimidine biosynthesis pathway and should help in the development of drugs amenable to the clinic.

Most importantly, we characterized in detail the interactions between this new series of pyrimidine biosynthesis inhibitors, which were identified in an ISRE-luciferase screen, and the innate antiviral response. It has been previously shown that pyrimidine biosynthesis inhibitors are weak stimulators of ISGs when applied alone (8, 13–18), but these drugs boost the expression of ISGs in response to either extracellular or intracellular stimuli, such as IFN-β, RIG-I ligands, or viruses (8, 12, 13, 15, 18). Besides, pyrimidine biosynthesis inhibitors do not induce any type of IFNs when applied alone, and virus growth inhibition by these drugs seems to occur independent of IFN-α/β synthesis (8, 15, 16), despite some recent report showing no antiviral effects in Vero cells which are deleted for the IFN-α/β gene cluster (18). Here, we showed that IFN-β, IFN-λ1, and IFN-λ2/3 syntheses induced by the transfection of 5′-triphosphate RNA molecules are significantly amplified in DD778-treated cells. In MV-infected cells, DD778 also increased the expression of IFN-λ1 and IFN-λ2/3, which were produced at high levels in this system. It should be noted that cells were infected 9 h before the addition of DD778, thus allowing the accumulation of viral RNAs necessary to trigger the innate immune response before the antiviral state was settled. This was not always performed in previous reports and might account for the contradictory results obtained by others (6, 16). These new observations do not challenge the idea that the antiviral state induced by pyrimidine deprivation is essentially IFN independent. Indeed, it has been previously reported that chemical inhibitors of JAK1, which is signaling downstream of type I, II, and III IFN receptors, did not reverse the antiviral state induced by pyrimidine biosynthesis inhibitors (15). Our results, rather, expand the impact of pyrimidine deprivation on the innate antiviral response by documenting the consequences on cytokine secretion and cell-to-cell communication. Indeed, we showed that when cells are deprived of pyrimidines and suffer such a metabolic stress, the alarm signal they send to neighboring cells in response to RIG-I ligands is enhanced. Quite interestingly, this links cellular metabolism to cytokine exchanges and cell-to-cell communication in the context of innate immunity. However, the way in which pyrimidine biosynthesis inhibitors influence the expression of innate immunity genes remains a pending question. Although this is clearly a consequence of pyrimidine deprivation, since it is abolished when culture medium is supplemented with uridine, the mechanisms involved are still poorly understood. Most recently, it has been shown in Vero cells that RIG-I is among the ISGs induced by pyrimidine biosynthesis inhibitors, which could account for some enhanced sensing of 5′-triphosphate RNA (16). However, we did not confirm this observation in HEK-293 cells (Fig. 7A and B and 8B), suggesting that RIG-I induction by pyrimidine biosynthesis inhibitors alone is cell type dependent. In the future, a major challenge will be to identify the factors that sense intracellular levels of pyrimidines and the signaling pathways triggered to induce an antiviral state, to promote the expression of ISGs, and to enhance the synthesis of IFNs.

## MATERIALS AND METHODS

### Compound library, screening procedure, and DD363 analogs.

The initial screen that allowed the identification of DD363 was described in detail elsewhere (8). Briefly, HEK-293T cells were transfected with the pISRE-luciferase reporter plasmid (reference no. 219089; Stratagene) and dispensed at 2 × 10^4^ cells/well in white 96-well plates already containing 1 μl of compounds from the Institut Curie library at 2 mg/ml in DMSO. Since the final culture volumes were 100 μl/well, compounds were screened at 20 μg/ml, corresponding to 57 μM for DD363. Compound synthesis is described in in the supplemental material and Table 1.

### Cell lines, culture medium, and luciferase assays.

Cells were cultured at 37°C and 5% CO_2_ in Dulbecco's modified Eagle's medium (DMEM; Gibco-Invitrogen) containing 10% fetal calf serum (FCS), penicillin, and streptomycin. Human MRC5 and HEK-293T cells were from the ATCC. The reporter cell line STING-37, corresponding to HEK-293 cells stably transfected with the ISRE-luciferase reporter gene, was previously described (8). Luciferase induction in STING-37 cells was determined using the Bright-Glo (Promega) or Britelite plus reagents (PerkinElmer), according to the manufacturer's recommendations. Bioluminescence was measured for 0.1 s with a luminometer (EnSpire; PerkinElmer). Cellular viability was determined by quantification of ATP in culture wells using the CellTiter-Glo assay (Promega).

### Viruses, infections, and immunostaining.

Experiments with measles virus (MV) were performed with the vaccine strain Schwarz from the ATCC. The recombinant MV strain expressing firefly luciferase (MV-Luc) from an additional transcription unit was derived from the vaccine strain Schwarz and was previously described (30). Virus stocks were produced on Vero cells and titrated by 50% tissue culture infective dose (TCID_50_) on Vero cells. Chikungunya virus (CHIKV) infections were performed with wild-type strain 05115 from La Réunion Island or the recombinant strain CHIKV/Ren expressing Renilla luciferase (kindly provided by Philippe Desprès [19]). HCoV-229E was originally obtained from the ATCC (VR-740), and virus stocks were produced on MRC5 cells, as previously described (31). Firefly and Renilla luciferase expression from MV-Luc and CHIKV/Ren recombinant viruses was determined using the Bright-Glo (Promega) and Renilla-Glo (Promega) reagents, respectively.

For immunofluorescence detection of viral proteins, HEK-293T cells were infected with CHIKV at an MOI of 0.1 and cultured on microscopy slides (Ibidi, Munich, Germany). At 24 h postinfection, cells were fixed for 15 min with 4% paraformaldehyde (PFA) and permeabilized for 5 min at 4°C in phosphate-buffered saline (PBS) containing 0.1% Triton X-100. Cells were incubated for 1 h in PBS supplemented with 5% goat serum and incubated again in the same solution containing the Cy3-conjugated antibody against the CHIKV E2 protein (clone 3E4; kindly provided by Philippe Desprès) (32). For HCoV-229E, MRC5 cells were infected at an MOI of 0.1 and cultured on microscopy slides (Ibidi) for 48 h. Cells were fixed and permeabilized as described above and stained with an anti-spike glycoprotein monoclonal antibody (5-11H.6; kindly provided by Pierre Talbot and Marc Desforges, INRS-Institut Armand Frappier, Canada), which was detected with a Cy3-conjugated goat anti-mouse IgG (no. 115-166-072; Jackson ImmunoResearch). Finally, cells were washed with PBS and stained for 5 min with a PBS solution containing 4′,6-diamidino-2-phenylindole (DAPI). After washing in PBS, 100 μl of Fluoromount-G was added in the wells (SouthernBiotech, Birmingham, AL, USA). The slides were analyzed, and image acquisition was performed using fluorescence microscope using a 40× oil immersion objective.

### Chemical reagents, recombinant IFN-β, and ssRNA synthesis.

Uridine, orotate, dihydroorotate, brequinar sodium salt, and dicoumarol were from Sigma-Aldrich. IFN-β (11410-2) was from PBL Biomedical Laboratories. Short synthetic 5′-triphosphate RNA molecules (ssRNA) were synthesized by *in vitro* transcription using the T7 RiboMAX Express large-scale RNA production system (Promega), and the pCI-neo vector was digested with XbaI as the DNA template. The T7 transcription start is located 36 nucleotides upstream of the Xba1 restriction site, but runoff transcription *in vitro* is known for producing at a high yield longer 5′-triphosphate RNA molecules that include copy-back sequences and fold into stem-loop structures (33). Such ssRNA molecules were previously shown to efficiently stimulate the interferon response by the RIG-I/MAVS pathway when transfected in HEK-293 or STING-37 cells (8, 12, 22, 34). For each production lot, pools of *in vitro* transcripts were purified with a filtering membrane (SEQ96 sequencing reaction cleanup; Millipore), analyzed on a Bioanalyzer RNA Nano kit (Agilent), and qualified for their immunostimulatory properties on STING-37 reporter cells. ssRNA transfections were performed with JetPrime PEI, according to the manufacturer's recommendations (Polyplus-transfection). Briefly, 1 μg of RNA was mixed with 100 μl of commercial buffer and 2 μl of JetPrime reagent and incubated 20 min at room temperature before proceeding to transfection. A total of 2 × 10^5^ cells were transfected with either 60 or 120 ng of ssRNA, as indicated in the figures (Fig. 6A and C and 7A to C; see also Fig. S2B and S3D).

### Metabolite analyses.

HEK-293T cells were plated in 6-well plates at 1 × 10^6^ cells per well. After 24 h, culture medium was supplemented with DD778 or DMSO alone. Cells were harvested 1 day later and washed with PBS. All extraction steps were performed on ice. Cellular pellets were deproteinized with an equal volume of 6% perchloric acid (PCA), vortex mixed for 20 s, ice-bathed for 10 min, and vortex mixed again for 20 s. Acid cell extracts were centrifuged at 13,000 rpm for 10 min at 4°C. The resulting supernatants were supplemented with an equal volume of bidistilled water, vortex mixed for 60 s, and neutralized by the addition of 2 M Na_2_CO_3_. Extracts were injected onto a C_18_ Supelco 5-μm (250 by 4.6 mm) column (Sigma) at 45°C. The mobile phase was delivered at a flow rate of 1 ml/min using the following stepwise gradient elution program: A to B at 60:40 at 0 min, 40:60 at 30 min, and 40:60 at 60 min. Buffer A contained 10 mM tetrabutylammonium hydroxide, 10 mM KH_2_PO_4_, and 0.25% MeOH and was adjusted to pH 6.9 with 1 M HCl. Buffer B consisted of 5.6 mM tetrabutylammonium hydroxide, 50 mM KH_2_PO_4_, and 30% MeOH and was neutralized to pH 7.0 with 1 M NaOH. Detection was done with a diode array detector (PDA). The LC Solution workstation chromatography manager was used to pilot the HPLC instrument and to process the data. Products were monitored spectrophotometrically at 254 nm and quantified by integration of the peak absorbance area, employing a calibration curve established with various known nucleosides. Finally, a correction coefficient was applied to correct raw data for minor differences in the total number of cells determined under each culture condition.

### Gene expression analysis by RT-qPCR.

The transcription levels of type I IFN genes and ISGs presented in Fig. 7 were determined by RT-qPCR. Total RNA was extracted from 2 × 10^5^ cells using the RNeasy Micro kit and submitted to DNase treatment (Qiagen), according to the manufacturer's instructions. RNA was converted to cDNA with the RevertAid H Minus first-strand cDNA synthesis kit (Thermo Scientific). Real-time PCRs were performed in duplicate using Takyon ROX SYBR MasterMix blue dTTP (Eurogentec) on a 7900HT Fast real-time PCR system (Applied Biosystems). Transcripts were quantified using the following program: 3 min at 95°C, followed by 35 cycles of 15 s at 95°C, 25 s at 60°C, and 25 s at 72°C. Values for each transcript were normalized to the expression levels of RPL13A (60S ribosomal protein L13a) using the 2^−ΔΔ*CT*^ method. The primers used for the quantification of transcripts are the following: RPL13A-F (CCTGGAGGAGAAGAGGAAAGAGA) and RPL13A-R (TTGAGGACCTCTGTGTATTTGTCAA), RIG-I-F (ATCCAAACCAGAGGCAGAGGAA) and RIG-I-R (ACTGCTTCGTCCCATGTCTGAA), MDA5-F (ACAGCTTCACCTGGTGTTGGAG) and MDA5-R (CTTGCATGGCTCCTGTATTTGG), OASL-F (TCGTGAAACATCGGCCAACT) and OASL-R (AAGAGCATAGAGAGGGGGCA), IFN-α4-F (CCCACAGCCTGGGTAATAGGA) and IFN-α4-R (CAGCAGATGAGTCCTCTGTGC), IFN-β-F (TGCATTACCTGAAGGCCAAGG) and IFN-β-R (AGCAATTGTCCAGTCCCAGAG), IFN-γ-F (GGCAGCCAACCTAAGCAAGAT) and IFN-γ-R (CAGGGTCACCTGACACATTCA), IFN-λ1-F (GGACGCCTTGGAAGAGTCAC) and IFN-λ1-R (CTGGTCTAGGACGTCCTCCA), and IFN-λ2/3-F (GGGCCTGTATCCAGCCTCAG) and IFN-λ2/3-R (GAGGAGGCGGAAGAGGTTGA).

### Western blot analysis.

A total of 2 × 10^5^ STING-37 cells were left untreated or were transfected with 60 or 120 ng of ssRNA using the JetPrime transfection reagent (see above) and cultured in a 24-well plate with DD778 at 30 μM or DMSO alone. Alternatively, cells were infected with MV (MOI = 2) and cultured for 9 h before the addition of DD778 or DMSO alone. At T = 48 h, cells were washed in PBS and resuspended in RIPA lysis buffer (1% Nonidet P-40, 0.5% Na-deoxycholate, 0.1% SDS, 50 mM Tris-HCl [pH 7.4], 150 mM NaCl, 2 mM EDTA, and 50 mM NaF) supplemented with cOmplete protease inhibitor cocktail (Roche). After homogenization through a needle and incubation on ice for 20 min, cell lysates were clarified by centrifugation at 14,000 × *g* for 20 min. Protein extracts were resolved by SDS-polyacrylamide gel electrophoresis (SDS-PAGE) on 4 to 12% NuPAGE Bis-Tris gels with morpholinepropanesulfonic acid (MOPS) running buffer (Thermo Fisher) and transferred to a nitrocellulose membrane. Proteins were detected using standard immunoblotting techniques using the following primary antibodies: anti-MDA5 rabbit polyclonal antibody (clone AT113) and anti-RIG-I mouse monoclonal antibody (clone Alme1), from Alexis Biochemicals, and anti-β-actin mouse monoclonal antibody, from Sigma-Aldrich (clone AC-15, catalog no. A5441). Secondary anti-mouse and anti-rabbit horseradish peroxidase (HRP)-conjugated antibodies were from Dako (catalog no. P0447) and Sigma-Aldrich (catalog no. A0545), respectively. Protein detection was performed using the SuperSignal West Pico chemiluminescent substrate (Thermo Fisher Scientific) and quantified with the Adobe Photoshop software.

### Cytokine quantification in culture supernatants.

Cytokine levels in culture supernatants of HEK-293 cells stimulated with ssRNA or MV with or without DMSO or DD778 at 30 μM were determined using the LEGENDplex analysis kit, according to the manufacturer's recommendations (human anti-virus response panel with V-bottom plate, catalog no. 740390).

## Supplementary Material

Supplemental material
