# Supplementary material for: Original Chemical Series of Pyrimidine Biosynthesis Inhibitors That Boost the Antiviral Interferon Response
Source: Antimicrob Agents Chemother. 2017 Sep 22;61(10):e00383-17. doi: 10.1128/AAC.00383-17 (PMC5610480; doi:10.1128/AAC.00383-17)
Supplement: Supplemental material [file supp_61_10_e00383-17__index.html]

Supplemental material 

# Original Chemical Series of Pyrimidine Biosynthesis Inhibitors That Boost the Antiviral Interferon Response

## Supplemental material

- Supplemental file 1 -

  Fig. S1, Fig. S2, Fig. S3, Text S1

  PDF, 456K
