# Supplementary material for: Original Chemical Series of Pyrimidine Biosynthesis Inhibitors That Boost the Antiviral Interferon Response
Source: Antimicrob Agents Chemother. 2017 Sep 22;61(10):e00383-17. doi: 10.1128/AAC.00383-17 (PMC5610480; doi:10.1128/AAC.00383-17)
Supplement: Supplemental material [file AAC.00383-17_zac010176596s1.pdf]

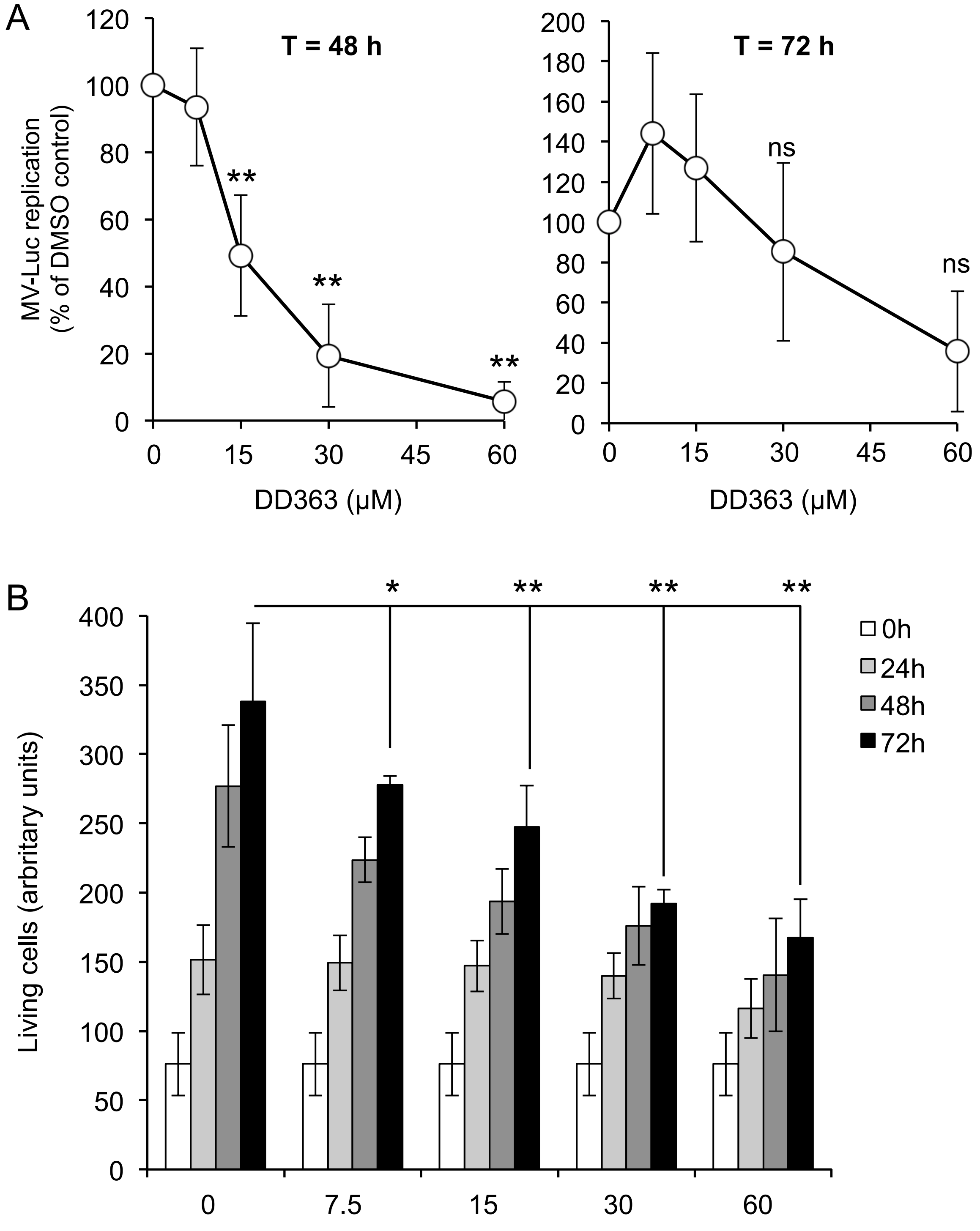

FIG. S1. Kinetic study of DD363 effects on MV growth and cellular proliferation. (**A**) HEK-293T cells were infected with MV-Luc (MOI = 0.1), and incubated with increasing concentrations of DD363 or DMSO alone. After 48 or 72 h of culture, luciferase activity was measured to quantify viral growth. Results are expressed as a percentage of luminescence signals relative to DMSO control (means ± SD of 5 and 3 independent experiments, respectively). ** $P<0.01$ as calculated by one-way ANOVA with Bonferroni's post hoc test. "ns" for "non significant". (**B**) HEK-293T cells were incubated with increasing doses of DD363 or DMSO alone. After 0, 24, 48 and 72 h of culture, the number of living cells was determined using CellTiter-Glo reagent. The number of living cells is expressed as a percentage relative to the initial number of living cells at T=0 h. * $P<0.05$ and ** $P<0.01$ as calculated by two-way ANOVA with Bonferroni's post hoc test.

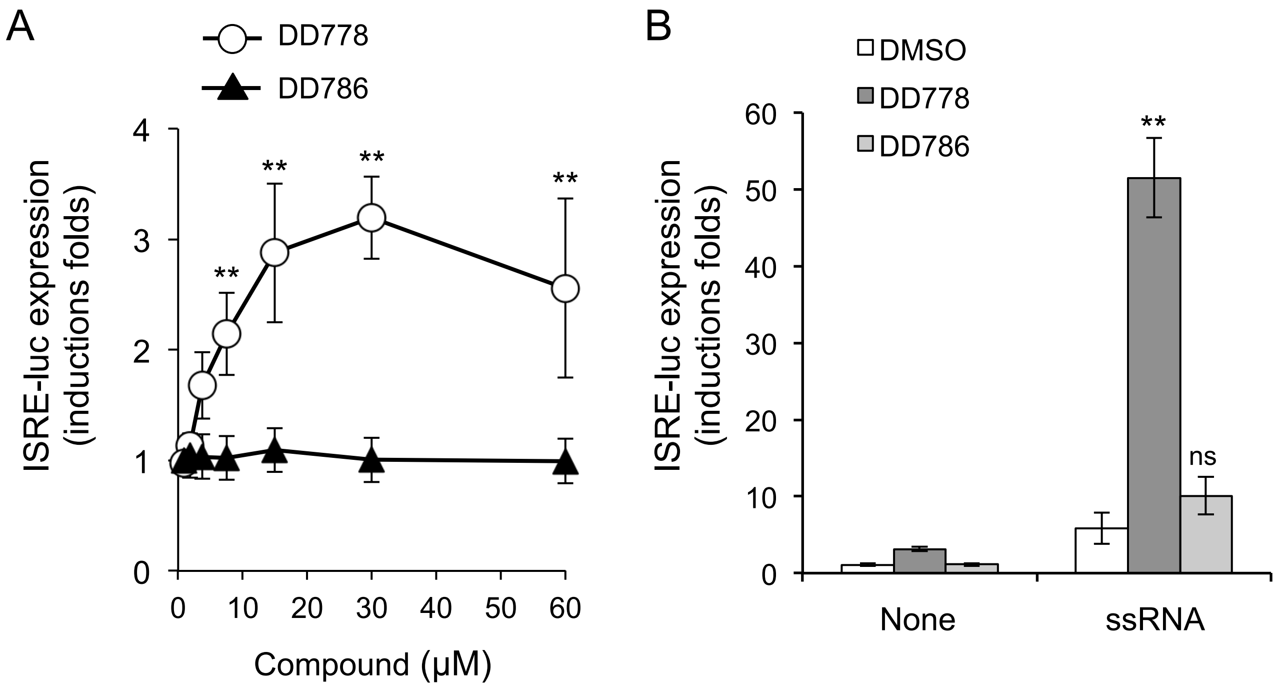

FIG. S2. As opposed to DD778, the analog DD786 had no effect on the ISRE-luciferase reporter gene, either alone or when combined to ssRNA transfection. (A) STING-37 reporter cells were incubated with increasing doses of DD778, DD786 or DMSO alone. After 24 h, luciferase expression was determined and results expressed as a fold-change relative to DMSO control. Data correspond to means ± SD of 3 independent experiments. ** $P<0.01$ as calculated by one-way ANOVA with Bonferroni's post hoc test. **(B)** STING-37 cells were transfected with ssRNA (60 ng) and treated with DMSO, DD778 (30 µM) or DD786 (30 µM). After 24 h, luciferase expression was determined and results were expressed as a fold-change relative to DMSO control. Data correspond to means ± SD of 3 independent experiments. ** $P<0.01$ as calculated by two-way ANOVA with Bonferroni's post hoc test.

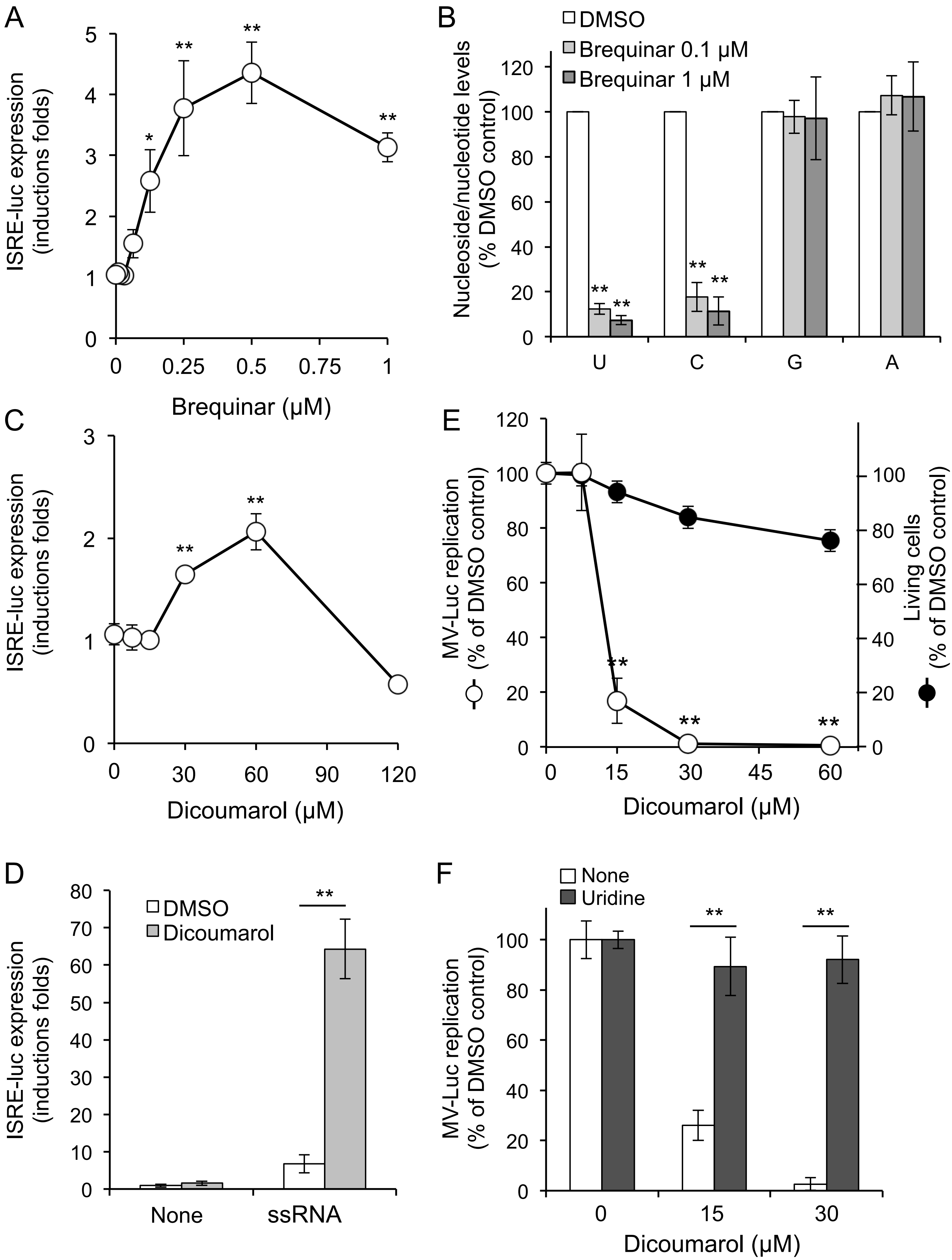

A
ISRE-luc expression (inductions folds)
Brequinar (µM)
B
DMSO
Brequinar 0.1 µM
Brequinar 1 µM
Nucleoside/nucleotide levels (% DMSO control)
U
C
G
A
C
ISRE-luc expression (inductions folds)
Dicoumarol (µM)
E
MV-Luc replication (% of DMSO control)
Living cells (% of DMSO control)
Dicoumarol (µM)
D
DMSO
Dicoumarol
ISRE-luc expression (inductions folds)
None
ssRNA
F
None
Uridine
MV-Luc replication (% of DMSO control)
Dicoumarol (µM)

FIG. S3. (A) STING-37 cells were incubated with increasing doses of brequinar. After 24 h, luciferase expression was determined and results expressed as a fold-change relative to untreated cells. **(B)** Cells were treated for 24 h with brequinar or DMSO alone, and intracellular levels of each nucleoside/nucleotide (U, C, G or A) were determined by HPLC and spectrophotometry. Concentrations are expressed as a percentage relative to DMSO-treated cells. **(C)** STING-37 cells were incubated with increasing doses of dicoumarol. After 24 h, luciferase expression was determined and results expressed as a fold-change relative to untreated cells. **(D)** STING-37 cells were transfected with ssRNA (60 ng) and treated with DMSO or dicoumarol (60 μM). After 24 h, luciferase expression was determined and results expressed as a fold-change relative to untreated cells. **(E)** HEK-293T cells were infected with MV-Luc (MOI = 0.1), and incubated with increasing concentrations of dicoumarol or DMSO alone. After 24 h, luciferase activity was measured to quantify viral growth (open circles). As a control, HEK-293T cells were treated with increasing concentrations of dicoumarol or DMSO alone. After 24 h, cellular viability was determined using CellTiter-Glo reagent (Promega; closed circles). Results are expressed as a percentage relative to DMSO control. **(F)** HEK-293T cells were infected with MV-Luc (MOI = 0.1), and incubated with DMSO or dicoumarol in the presence of uridine (130 μM) or not. After 24 h, luciferase expression that reflects viral growth was determined. All data from **A-F** correspond to means ± SD of 3 independent experiments, except for (D) that corresponds to 4 independent experiments. * $P<0.05$ and ** $P<0.01$ as calculated by one-way ANOVA with Bonferroni's post hoc test.

SUPPLEMENTARY MATERIAL

CHEMISTRY

Analysis: Melting points were measured on a Köfler hot stage apparatus and are uncorrected. Infrared spectra were recorded on a Perkin-Elmer RX I spectrometer as deuteriochloroform solutions (or KBr discs) or with a Nicolet Magna 550 FTIR spectrometer fitted with a horizontal Attenuated Total Reflectance (ATR) Durascope Durasampler equipped with a diamond / KRS5 internal reflection element; a DTGS detector was used at a resolution of $4cm^{-1}$. The ^{1}H-NMR (300 MHz) spectra were recorded on a Varian AC 300 spectrometer. Chemical shifts are expressed as parts per million downfield from tetramethylsilane. Splitting patterns have been designated as follows: s (singlet), d (doublet), dd (doublet of doublet), t (triplet), dt (doublet of triplet), q (quadruplet), m (multiplet), br. (broad signal). Coupling constants (*J* values) are listed in hertz (Hz). Mass spectra were obtained with a ZQ 2000 MS spectrometer applying an electrospray $(ES)^{+}$ ionization technique.

* * *

- The 3-(3-chlorophenyl)-2-nitro-4*H*-furo[3,2-*c*][1]benzopyran-4-one **DD311** has already been described by one of us (1). The synthesis of the 3-(3-chlorophenyl)-6,7-dihydrobenzofuran-4(5*H*)-one **DD264** has previously been described (2). The other compounds tested herein were especially prepared for the present work. The new nitro derivatives **DD363**, **DD771, DD775, DD776, DD778**, **DD783, DD784, JP10, JP25, JP49, JP52,** were synthesized in their racemic forms starting from the appropriate 4-hyroxycoumarin and of the suitable 3-(2-chloro-2-nitrovinyl)-1*H*-indole by adapting a methodology previously reported involving potassium fluoride in refluxing 1,2-dimethoxyethane (Table 1)(1, 3).

General procedure : A 50-mL round-bottomed flask equipped with reflux condenser and a magnetic stirring bar was charged with the appropriate 4-hyroxycoumarin (10 mmol), anhydrous potassium fluoride (0.58g, 10 mmol) and the suitable 3-(2-chloro-2-nitrovinyl)-1*H*-indole (5 mmol) in 1,2-dimethoxyethane (25 mL). The mixture was refluxed with stirring under argon atmosphere for 30 hours. Removal of the solvent at 50°C under vacuum afforded a solid which was filtered and washed successively with dichloromethane then methanol.

3-(1H-indol-3-yl)-2-nitro-2H-furo[3,2-c]chromen-4(3H)-one **DD363**

Yield 79%; mp 249-251°C (dec.) recrystallized from toluene.

IR ν_{max} (cm^{-1}): 3363, 1713, 1656, 1561, 1370, 1080, 1069.

^{1}H NMR (DMSO-d6) δ (ppm): 5.38 (br. s, 1H); 6.96 (d, 1H, J = 2.0 Hz); 7.06 (t, 1H, J = 7.4 Hz); 7.14 (t, 1H, J = 7.4 Hz); 7.33 (d, 1H, J = 2.4 Hz); 7.40 (d, 1H, J = 8.1 Hz); 7.52 (t, 1H, J = 7.4 Hz); 7.59 (t, 1H, J = 6.8 Hz); 7.60 (s, 1H); 7.81 (dt, 1H, J = 1.5 Hz and 7.7 Hz); 7.92 (dd, 1H, J = 1.5 Hz and 7.7 Hz); 11.42 (br. s, 1H).

MS (*m/z*): 371 $[M + Na]^+$.

The two enantiomers of the racemic **DD 363** were separated at 25°C by normal phase HPLC on a chiral column (Chiralpak® IB (250 x 4,6 mm); Chiral Technologies, Illkirch France) eluting with ethanol as the mobile phase at a flow rate of 1 ml/min, coupled to a polarimeter. The detection wavelength was 254 nm. The two enantiomers, eluting at 4.71 min (+) and 7.71 min (-), respectively, were obtained satisfactorily pure on a 100 mg scale of each enantiomer after evaporation of the solvent (3).

***8-chloro-3-(1H-indol-3-yl)-2-nitro-2H-furo[3,2-c]chromen-4(3H)-one* DD771**

Yield 76 %; mp >260°C (dec.) recrystallized from a mixture toluene/acetonitrile.

IR ν_{max} (cm^{-1}): 3399, 1715, 1654, 1562, 1431, 1390, 1105, 1025.

^{1}H NMR (DMSO-d6) δ (ppm): 5.41 (d, 1H, J = 1.8 Hz); 6.98 (d, 1H, J = 1.8 Hz); 7.07 (dt, 1H, J = 1.0 and 7.5 Hz); 7.33 (d, 1H, J = 2.4 Hz); 7.35 (d, 1H, J = 2.4 Hz); 7.40 (d, 1H, J = 8.0 Hz); 7.61 (d, 1H, J = 7.6 Hz); 7.63 (d, 1H, J = 8.9 Hz); 7.85 (dd, 1H, J = 2.5 Hz and 8.9 Hz); 7.95 (d, 1H, J = 2.4 Hz); 11.23 (s, 1H).

MS (*m/z*): 383-385 $[M + H]^+$. 405-407 $[M + Na]^+$.

The two enantiomers of the racemic **DD 771** were separated at 25°C by normal phase HPLC on a chiral column (Chiralpak® IB (250 x 4,6 mm); Chiral Technologies, Illkirch France) eluted with an isocratic mobile phase ethanol/chloroform 8/2 (v/v) at a flow rate of 1 ml/min. The detection wavelength was 254 nm. The two enantiomers, eluting at 4.80 min (+) and 10.92 min (-), respectively, were collected to provide satisfactorily pure compounds (about 100 mg of each) after evaporation of the solvents (3).

***3-(1H-indol-3-yl)-7,8-dimethyl-2-nitro-2H-furo[3,2-c]chromen-4(3H)-one* DD775**

Yield 81 %; mp >260°C (dec.) recrystallized from a mixture toluene/acetonitrile.

IR ν_{max} (cm^{-1}): 3419, 1718, 1658, 1570, 1422, 1367, 1028, 1003.

^{1}H NMR (DMSO-d6) δ (ppm): 2.35 (s, 3H); 2.37 (s, 3H); 5.33 (br. s, 1H); 6.95 (br. s, 1H); 7.04 (t, 1H, J = 7.6 Hz); 7.13 (t, 1H, J = 7.2 Hz); 7.30 (br. s, 1H); 7.39 (d, 1H, J = 6.4 Hz); 7.41 (s, 1H); 7.53 (d, 1H, J = 7.6 Hz); 7.68 (s, 1H); 11.22 (s, 1H).

MS (*m/z*): 399 $[M + Na]^+$.

8-fluoro-3-(1H-indol-3-yl)-2-nitro-2H-furo[3,2-c]chromen-4(3H)-one **DD776**

Yield 78 %; mp 233-235°C (dec.) recrystallized from a mixture toluene/acetonitrile.

IR ν_{max} (cm^{-1}): 3413, 1722, 1659, 1573, 1450, 1366, 1200, 1065, 1025.

^1H NMR (DMSO-d6) δ (ppm): 5.41 (d, 1H, J = 1.8 Hz); 6.99 (d, 1H, J = 1.8 Hz); 7.07 (t, 1H, J = 7.4 Hz); 7.15 (t, 1H, J = 7.2 Hz); 7.35 (d, 1H, J = 2.4 Hz); 7.46 (d, 1H, J = 7.9 Hz); 7.61 (d, 1H, J = 7.8 Hz); 7.63 - 7.80 (m, 3H); 11.23 (s, 1H).

MS (*m/z*): 389 $[M + Na]^+$.

3-(1H-indol-3-yl)-8-methyl-2-nitro-2H-furo[3,2-c]chromen-4(3H)-one **DD778**

Yield 77%; mp 254-256°C (dec.) recrystallized from a mixture toluene/acetonitrile.

IR ν_{max} (cm^{-1}): 3409, 1720, 1658, 1569, 1457, 1368, 1220, 1082, 1069, 1007

^1H NMR (DMSO-d6) δ (ppm): 2.47 (s, 3H); 5.39 (br. s, 1H); 6.98 (br. s, 1H); 7.07 (t, 1H, J = 7.4 Hz); 7.16 (t, 1H, J = 7.5 Hz); 7.34 (d, 1H, J = 2.3 Hz); 7.42 (d, 1H, J = 8.0 Hz); 7.49 (d, 1H, J = 8.5 Hz); 7.61 (d, 1H, J = 8.3 Hz); 7.63 (d, 1H, J = 9.0 Hz); 7.74 (s, 1H); 11.24 (s, 1H).

MS (*m/z*): 363 $[M + H]^+$. 385 $[M + Na]^+$.

3-(5-bromo-1H-indol-3-yl)-8-chloro-2-nitro-2H-furo[3,2-c]chromen-4(3H)-one **DD783**

Yield 74 %; mp 258-260°C (dec.) with allotropic change at 192-198°C recrystallized from toluene.

IR ν_{max} (cm^{-1}): 3427, 1722, 1652, 1570, 1368, 1299, 1103, 1024.

^1H NMR (DMSO-d6) δ (ppm): 5.47 (d, 1H, J = 1.8 Hz); 7.00 (d, 1H, J = 1.9 Hz); 7.27 (dd, 1H, J = 1.8 Hz and 8.6 Hz); 7.39 (d, 1H, J = 8.7 Hz); 7.41 (d, 1H, J = 2.0 Hz); 7.64 (d, 1H, J = 9.0 Hz); 7.85 (dd, 1H, J = 2.5 and 9.1 Hz); 7.87 (s, 1H); 7.94 (d, 1H, J = 2.5 Hz); 11.44 (s, 1H).

MS (*m/z*): 483-485-487 $[M + Na]^+$.

3-(5-bromo-1H-indol-3-yl)-8-methyl-2-nitro-2H-furo[3,2-c]chromen-4(3H)-one **DD784**

Yield 72 %; mp >260°C (dec.) recrystallized from chloroform.

IR ν_{max} (cm^{-1}): 3342, 1709, 1658, 1575, 1362, 1310, 1219, 1087, 1011.

^1H NMR (DMSO-d6) δ (ppm): 2.45 (s, 3H); 5.42 (d, 1H, J = 1.5 Hz); 6.99 (d, 1H, J = 1.9 Hz); 7.26 (dd, 1H, J = 1.7 Hz and 8.6 Hz); 7.36 (br. s, 1H); 7.38 (d, 1H, J = 8.7 Hz); 7.48 (d, 1H, J = 8.5 Hz); 7.62 (dd, 1H, J = 1.7 and 8.6 Hz); 7.71 (s, 1H); 7.83 (d, 1H, J = 1.4 Hz); 11.43 (s, 1H).

MS (*m/z*): 441-443 $[M + H]^+$, 463-465 $[M + Na]^+$.

***8-chloro-3-(1-methyl-1H-indol-3-yl)-2-nitro-2H-furo[3,2-c]chromen-4(3H)-one* JP10**

Yield 66 %; mp >260°C (dec.) recrystallized from toluene.

IR ν_{max} (cm^{-1}): 1718, 1657, 1566, 1494, 1478, 1430, 1296, 1104, 1073, 1010.

^{1}H NMR (DMSO-d6) δ (ppm): 3.72 (s, 3H); 5.44 (d, 1H, J = 1.7 Hz); 6.98 (d, 1H, J = 1.9 Hz); 7.14 (dt, 1H, J = 0.9 Hz and 7.9 Hz); 7.24 (dt, 1H, J = 1.0 Hz and 7.6 Hz); 7.38 (s, 1H); 7.48 (d, 1H, J = 8.2 Hz); 7.66 (br. d, 2H, J = 9.0 Hz); 7.88 (dd, 1H, J = 2.5 Hz and 8.9 Hz); 7.97 (d, 1H, J = 2.5 Hz).

MS (*m/z*): 397-399 $[M + H]^+$, 419-421 $[M + Na]^+$.

***8-chloro-3-(2-methyl-1H-indol-3-yl)-2-nitro-2H-furo[3,2-c]chromen-4(3H)-one* JP25**

Yield 71 %; mp 191-192°C (dec.) recrystallized from a mixture toluene/acetonitrile.

IR ν_{max} (cm^{-1}): 3347, 1726, 1657, 1570, 1461, 1384, 1287, 1102, 1031.

^{1}H NMR (DMSO-d6) δ (ppm): 2.43 (s, 3H); 5.42 (d, 1H, J = 2.7 Hz); 6.86 (t, 1H, J = 7.5 Hz); 7.00 (t, 1H, J = 7.8 Hz); 7.02 (t, 1H, J = 7.2 Hz); 7.08 (d, 1H, J = 2.6 Hz); 7.29 (d, 1H, J = 7.9 Hz); 7.60 (d, 1H, J = 9.0 Hz); 7.84 (dd, 1H, J = 2.5 Hz and 9.0 Hz); 8.02 (d, 1H, J = 2.5 Hz); 11.20 (s, 1H).

MS (*m/z*): 397-399 $[M + H]^+$, 419-421 $[M + Na]^+$.

***8-ethyl-3-(1H-indol-3-yl)-2-nitro-2H-furo[3,2-c]chromen-4(3H)-one* JP49**

Yield 79 %; mp 253-255°C (dec.) recrystallized from a mixture heptane/acetonitrile.

IR ν_{max} (cm^{-1}): 3405, 1716, 1663, 1565, 1458, 1419, 1369, 1219, 1072, 1024.

^{1}H NMR (DMSO-d6) δ (ppm): 1.26 (t, 3H, J = 7.6 Hz); 2.78 (q, 2H, J = 7.6 Hz); 5.39 (d, 1H, J = 1.7 Hz); 6.98 (d, 1H, J = 1.9 Hz); 7.08 (dt, 1H, J = 1.0 Hz and 8.0 Hz); 7.16 (dt, 1H, J = 1.0 Hz and 8.0 Hz); 7.34 (d, 1H, J = 2.4 Hz); 7.42 (d, 1H, J = 8.0 Hz); 7.52 (d, 1H, J = 8.6 Hz); 7.61 (d, 1H, J = 7.8 Hz); 7.68 (dd, 1H, J = 2.1 Hz and 8.6 Hz); 7.74 (d, 1H, J = 1.9 Hz); 11.23 (s, 1H).

MS (*m/z*): 399 $[M + Na]^+$.

***8-bromo-3-(1H-indol-3-yl)-2-nitro-2H-furo[3,2-c]chromen-4(3H)-one* JP52**

Yield 70 %; mp > 260°C (dec.) recrystallized from a mixture toluene/acetonitrile.

IR ν_{max} (cm^{-1}): 3401, 1718, 1654, 1557, 1456, 1427, 1366, 1292, 1220, 1091, 1068, 1023.

^{1}H NMR (DMSO-d6) δ (ppm): 5.43 (d, 1H, J = 1.6 Hz); 6.99 (d, 1H, J = 1.9 Hz); 7.08 (br. t, 1H, J = 7.1 Hz); 7.16 (br. t, 1H, J = 7.1 Hz); 7.37 (d, 1H, J = 2.3 Hz); 7.42 (d, 1H, J = 8.0 Hz); 7.58 (d, 1H, J = 8.9 Hz); 7.61 (br. d, 1H, J = 7.8 Hz); 7.98 (dd, 1H, J = 2.4 Hz and 8.9 Hz); 8.07 (d, 1H, J = 2.3 Hz); 11.25 (s, 1H).

MS (*m/z*): 449-451 $[M + Na]^+$.

The two non-nitrated derivatives **JP42** and **JP46** were prepared in their racemic forms starting from the appropriate 4-hydroxycoumarine (8 mmol) and 3-(2-nitrovinyl)-1*H*-indole (4 mmol) by heating the mixture at 80°C in dimethylsulfoxide (25 mL) for 16 hours in the presence of potassium carbonate (2.5 mmol). The reaction was monitored by TLC (eluent hexane/ethyl acetate 8/2). After evaporation of the solvent, the obtained material was taken up with dichloromethane. The solution was washed three times with water and the organic layer was dried (MgSO4). Filtration, followed by evaporation of the solvent under vacuum, provided a crude product which was chromatographed on silicagel (100g, eluent hexane/ethyl acetate 1/1) to afford the wanted product which was further recrystallized (Scheme 1).

3-(1H-indol-3-yl)-2H-furo[3,2-c]chromen-4(3H)-one **JP42**

Yield 40 %; mp 160-161°C recrystallized from isopropanol.

IR ν_{max} (cm^{-1}): 3310, 1711, 1692, 1641, 1605, 1498, 1414, 1093, 1020.

^{1}H NMR (DMSO-d6) δ (ppm): 4.73 – 4.80 (m, 1H, C part of ABC system, J_{BC} = 5.5 Hz and J_{AC} = 9.0 Hz); 4.92 – 5.00 (m, 1H, B part of ABC system, J_{BC} = 5.5 Hz and J_{AB} = 10.1 Hz); 5.23 – 5.32 (m, 1H, A part of ABC system, J_{AC} = 9.0 Hz and J_{AB} = 10.1 Hz); 6;93 (br. t, 1H, J = 7.4 Hz); 7.07 (br. t, 1H, J = 7.5 Hz); 7.23 (d, 1H, J = 2.3 Hz); 7.34 – 7.52 (m, 4H); 7.72 (dt, 1H, J = 1.5 Hz and 8.5 Hz); 7.82 (dd, 1H, J = 1.3 Hz and 7.7 Hz); 8.70 (s, 1H); 10.98 (s, 1H).

MS (*m/z*): 326 $[M + Na]^+$.

8-chloro-3-(1H-indol-3-yl)-2H-furo[3,2-c]chromen-4(3H)-one **JP46**

Yield 42 %; mp 254-255°C (dec.) recrystallized from a mixture heptane/acetonitrile.

IR ν_{max} (cm^{-1}): 3394, 1715, 1642, 1421, 1397, 1299, 1266, 1223, 1106, 1018.

^{1}H NMR (DMSO-d6) δ (ppm): 4.73 – 4.80 (m, 1H, C part of ABC system, J_{BC} = 5.6 Hz and J_{AC} = 8.9 Hz); 4.92 – 5.01 (m, 1H, B part of ABC system, J_{BC} = 5.6 Hz and J_{AB} = 10.0 Hz); 5.23 – 5.32 (m, 1H, A part of ABC system, J_{AC} = 8.9 Hz and JAB = 10.0 Hz); 6;94 (br. t, 1H, J = 7.4 Hz); 7.08 (br. t, 1H, J = 7.6 Hz); 7.25 (d, 1H, J = 2.3 Hz); 7.38 (br. t, 2H, J = 7.6 Hz); 7.53 (d, 1H, J = 8.9 Hz); 7.75 (dd, 1H, J = 2.4 Hz and 8.9 Hz); 7.83 (d, 1H, J = 2.4 Hz); 11.01 (s, 1H).

MS (*m/z*): 360-362 $[M + Na]^+$.

In an attempt to prepare the 3-(1*H*-indol-3-yl)-2*H*-furo[3,2-*c*]chromen-4(3*H*)-one **JP42** in a better yield, the above reaction has been carried out at 50°C (instead of 80 °C) for 18 hours. Under these conditions, it

was possible to isolate a more polar compound characterized as the non-cyclized intermediate **JP39** (Scheme 2).

***3-(1-(1H-indol-3-yl)-2-nitroethyl)-4-hydroxy-2H-chromen-2-one* JP39**

Yield 21 %; mp 157-160°C (dec.) with allotropic change at 135-145°C recrystallized from benzene.

IR ν_{max} (cm^{-1}): 3406, 1673, 1607, 1546, 1377, 1206, 1095.

^{1}H NMR (DMSO-d6) δ (ppm): 5.29 – 5.38 (m, 1H, C part of ABC system, J_{BC} = 6.6 Hz and J_{AC} = 12.1 Hz); 5.43 – 5.59 (m, 2H, BC part of ABC system, J_{BC} = 6.6 Hz, J_{AB} = 8.6 Hz and J_{AC} = 12.1 Hz); 6;95 (dt, 1H, J = 1.0 Hz and J = 7.4 Hz); 7.03 (dt, 1H, J = 1.0 Hz and J = 7.5 Hz); 7.27 – 7.39 (m, 4H); 7.54 – 7.63 (m, 2H); 8.04 (dd, 1H, J = 1.1 Hz and 8.3 Hz); 10.98 (s, 1H); 11.80 – 12.50 (br. signal, 1H).

MS (*m/z*): 373 $[M + Na]^+$.

Several new 3-(2-nitro-2,3-dihydrofuran-3-yl)-1*H*-indole derivatives - structurally related to **DD363** and its furo[3,2-c]chromen-4(3H)-one congeners (*vide supra*) - were synthesized, under the same conditions, from the 3-(2-chloro-2-nitrovinyl)-1*H*-indole and the appropriate β-hydroxyketo starting materials. Thus, from 4-hydroxy-[1, 2]naphthoquinone, from 4-hydroxy-1-methyl-1H-quinolin-2-one or from 4-hydroxy-6-methyl-pyran-2-one, compounds **DD785**, **DD786** and **DD787** were respectively obtained (Scheme 3, 4 and 5).

***3-(1H-indol-3-yl)-2-nitro-2,3-dihydronaphtho[1,2-b]furan-4,5-dione* DD785**

Yield 64 %; mp 234-236°C (dec.) recrystallized from benzene.

IR ν_{max} (cm^{-1}): 3405, 1682, 1655, 1642, 1590, 1568, 1350, 1213, 1184, 1162, 1040, 1019.

^{1}H NMR (DMSO-d6) δ (ppm): 5.46 (br. s, 1H); 6.75 (br. s, 1H); 7.02 – 7.20 (m, 2H); 7.41 (br. s, 2H); 7.70 (d, 1H, J = 2.3 Hz); 7.80 – 7.97 (m, 3H); 8.09 (d, 1H, J = 2.6 Hz); 11.22 (s, 1H).

MS (*m/z*): 361 $[M + H]^+$, 383 $[M + Na]^+$.

***3-(1H-indol-3-yl)-6-methyl-2-nitro-2H-furo[3,2-c]pyran-4(3H)-one* DD786**

Yield 80 %; mp 229-230°C recrystallized from a mixture heptane chloroforme.

IR ν_{max} (cm^{-1}): 3368, 1712, 1585, 1568, 1447, 1423, 1372, 1357, 1232, 1092, 1052.

^{1}H NMR (DMSO-d6) δ (ppm): 3.32 (s, 3H); 5.15 (br. s, 1H); 6.76 (d, 1H, J = 1.9 Hz); 6.77 (s, 1H); 7.05 (br. t, 1H, J = 7.6 Hz); 7.14 (dt, 1H, J = 1.0 Hz and 8.0 Hz); 7.21 (d, 1H, J = 2.4 Hz); 7.40 (d, 1H, J = 8.0 Hz); 7.53 (d, 1H, J = 7.7 Hz); 11.19 (s, 1H).

MS (*m/z*): 313 $[M + H]^+$, 335 $[M + Na]^+$.

3-(1H-indol-3-yl)-5-methyl-2-nitro-2,3-dihydrofuro[3,2-c]quinolin-4(5H)-one **DD787**

Yield 71 %; mp 252-254°C (dec.) recrystallized from a mixture toluene/acetonitrile.

IR ($CDCl_3$) ν_{max} (cm^{-1}): 3380, 1671, 1639, 1600, 1562, 1459, 1421, 1371, 1238, 1100, 1070, 1039.

^{1}H NMR (DMSO-d6) δ (ppm): 3.59 (s, 3H); 5.26 (br. s, 1H); 6.88 (t, 1H, J = 1.5 Hz); 7.03 (br. t, 1H, J = 7.4 Hz); 7.09 – 7.18 (m, 2H); 7.40 (d, 1H, J = 7.6 Hz); 7.43 (t, 1H, J = 7.4 Hz); 7.56 (d, 1H, J = 7.8 Hz); 7.67 (d, 1H, J = 8.6 Hz); 7.79 (dt, 1H, J = 1.1 Hz and 7.3 Hz); 7.94 (d, 1H, J = 7.7 Hz); 11.15 (s, 1H).

MS (*m/z*): 384 $[M + Na]^+$.

REFERENCES

1. Dauzonne D, Josien H, Demerseman P. 1990. (2-Chloro-2-Nitroethenyl)Benzenes as Synthons - a General-Method for the Preparation of 2,3-Dihydro-2-Nitro-3-Phenyl-4h-Furo[3,2-C][1]Benzopyran-4-Ones and 3-Phenyl-4h-Furo[3,2-C][1]Benzopyran-4-Ones. Tetrahedron 46:7359-7371.

2. Lucas-Hourani M, Dauzonne D, Jorda P, Cousin G, Lupan A, Helynck O, Caignard G, Janvier G, Andre-Leroux G, Khiar S, Escriou N, Despres P, Jacob Y, Munier-Lehmann H, Tangy F, Vidalain PO. 2013. Inhibition of pyrimidine biosynthesis pathway suppresses viral growth through innate immunity. PLoS Pathog 9:e1003678.

3. Dauzonne D, Demerseman P. 1990. Potassium Fluoride-Promoted Reaction of (2-Chloro-2-Nitroethenyl)Benzenes with 1,3-Dicarbonyl Compounds - a General-Synthesis of 6,6-Dimethyl-2-Nitro-3-Phenyl-3,5,6,7-Tetrahydro-4(2h)Benzofuranones and Some Analogs. Journal of Heterocyclic Chemistry 27:1581-1584.

* * *

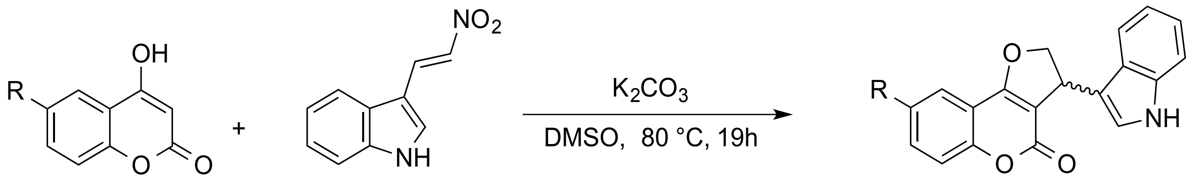

Scheme 1

**Product number**	R
**JP42** (*racemate*)	H
**JP46** (*racemate*)	Cl

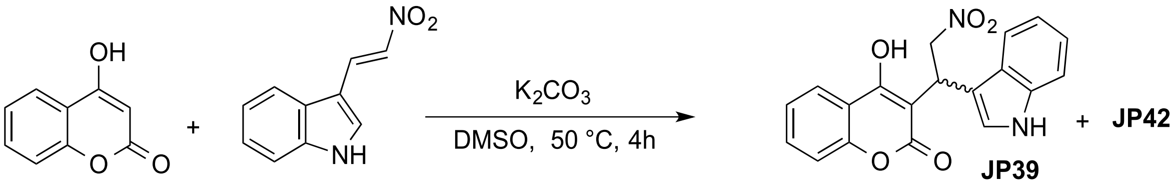

JP39
(*racemate*)

Scheme 2

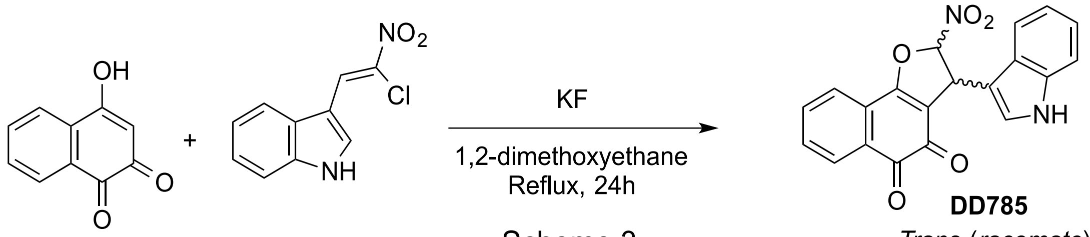

DD785
Trans (*racemate*)

Scheme 3

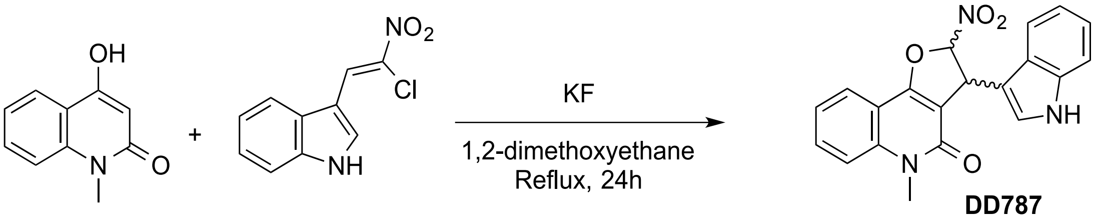

DD787
Trans (*racemate*)

Scheme 4

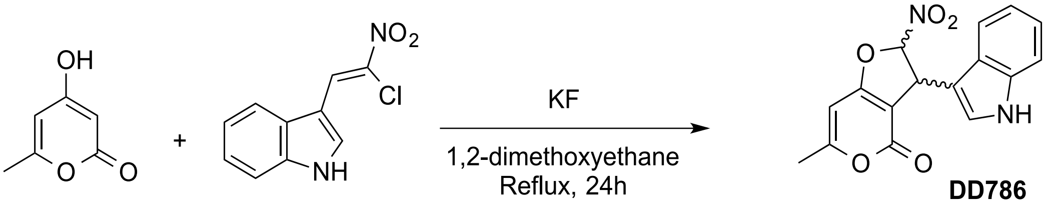

DD786
Trans (*racemate*)

Scheme 5
